# Genetic improvement of hip-extended scores in 3 breeds of guide dogs using estimated breeding values: Notable progress but more improvement is needed

**DOI:** 10.1371/journal.pone.0212544

**Published:** 2019-02-22

**Authors:** Eldin A. Leighton, Dolores Holle, Darryl N. Biery, Thomas P. Gregor, Mischa B. McDonald-Lynch, Mandy L. Wallace, Jennifer K. Reagan, Gail K. Smith

**Affiliations:** 1 The Seeing Eye, Inc, Morristown, New Jersey, United States of America; 2 Department of Clinical Sciences and Advanced Medicine, School of Veterinary Medicine, University of Pennsylvania, United States of America; University of Sydney Faculty of Veterinary Science, AUSTRALIA

## Abstract

Two hip quality phenotypes—a hip-extended score assigned by a board certified radiologist and the PennHIP distraction index—were analyzed to estimate genetic parameters and to calculate estimated breeding values used for selecting replacement breeders. Radiographs obtained at 12–18 months of age were available on 5,201 German Shepherd Dogs, 4,987 Labrador Retrievers and 2,308 Golden Retrievers. Obtained by fitting a two-trait model using Bayesian techniques, estimates of heritability for the hip-extended score were 0.76, 0.72, and 0.41 in German Shepherd Dogs, Labrador Retrievers, and Golden Retrievers, respectively, while estimated heritabilities for distraction index were 0.60, 0.66 and 0.59, respectively. Genetic correlations between the two hip quality measures were −0.28 in German Shepherd Dogs, −0.21 in Labrador Retrievers, and −0.29 in Golden Retrievers. Genetic selection for improved hip quality based upon the hip extended score phenotype began in 1980. Among first generation puppies, 34% of 273 German Shepherd Dogs, 55% of 323 Labrador Retrievers, and 43% of 51 Golden Retrievers had an Excellent hip extended score. After 8 generations of selection, mostly based on estimated breeding values derived from the hip extended score, over 93% of 695 German Shepherd Dogs, 94% of 528 Labrador Retrievers, and 87% of 116 Golden Retrievers received an Excellent hip extended score. With respect to PennHIP distraction index values among these same dogs, median values were at or above 0.30 for all 3 breeds meaning that half or more of dogs possessing the Excellent hip-extended-score phenotype remained susceptible to developing the osteoarthritis of canine hip dysplasia. Genetic improvement of the hip-extended-view phenotype to its desired biological endpoint left a surprising proportion of dogs expressing sufficient joint laxity to place them in an osteoarthritis at-risk state as they age. Only by directly applying selection pressure to reduce distraction index was marked reduction in joint laxity noted.

## Introduction

Canine hip dysplasia (CHD) is a highly prevalent orthopedic disease [1] of complex inheritance [2] [3] [4], meaning many genes and environmental factors [5] are responsible for phenotypic expression of the disorder. The disease phenotype is characterized by hip joint laxity in young dogs that, with age, often leads to development of secondary osteoarthritis (OA) [6] [7]. The pain and disability from OA of CHD is an animal welfare issue, and veterinarians have renewed their commitment to controlling CHD [8]. For working and service dogs, CHD can be extremely costly, profoundly shortening anticipated work longevity.

The OA of CHD that develops as a dog ages is the actual disease that dog breeders and veterinarians want to eliminate [6] [9] [10] [11]. Two principal strategies are available for controlling or preventing a physiologically complex trait like CHD [12]: genetic control or environmental (non-genetic) control. Genetic control aims to reduce CHD prevalence in future generations by selecting young breeding dogs possessing a genetic predisposition to produce offspring with reduced genetic susceptibility for developing CHD. This strategy requires an accurate hip screening test for use in individual dogs, and it requires utilizing time-proven animal breeding methods [13] to accurately identify exactly which young dogs are most likely to produce new offspring with low genetic risk of developing OA of CHD.

Environmental manipulation or non-genetic control strives to prevent, delay, or mitigate the expression of CHD in currently living dogs that carry risk of disease (e.g., pet and service dogs). Methods used include both surgical (eg, Juvenile Pubic Symphysiodesis, Triple Pelvic Osteotomy) [12] [14] and non-surgical (eg, weight control [15] [16], disease modifying osteoarthritic drugs, DMOADs)) strategies. Again, an accurate hip screening method, preferably applicable early in life, is necessary to identify dogs at risk of developing CHD, prior to implementing preventive measures.

### Radiographic approaches for evaluating hip quality

Physically, young dogs provide few conformational clues regarding their propensity to develop OA at later ages. Recognition of this fact led veterinarians and radiology specialists to develop radiographic images of hip joints in various positions [17] [18] [19] [20] [21]. Two of these positions have been used extensively: (a.) the ventrodorsal position, also known as the hip-extended view (HEV) [17], adapted for the dog from human hip radiography in 1961 [22], and (b.) the PennHIP distraction view [23] [6] position, first published in 1990.

Using radiographic images obtained from these two positions, three often used, but quite different phenotypes have been defined for describing CHD status of an individual dog. These phenotypes include: (a.) a subjective or qualitative score derived from the HEV position image, (b.) the Norberg angle (NA), a quantitative score, also derived from the HEV image, and (c.) the PennHIP distraction index (DI), a quantitative score derived from the PennHIP distraction view position. Because it was first to be introduced, the HEV position coupled with a qualitative score or grade derived from it became the “convention” for assessing hip quality throughout the world [4] [18] [24] [25] [26]. An extensive review of HEV-based schemes [4] provides details about their similarities and differences.

Research results, culminating 10 years of data collection, were first published in 1993 [6] showing that increased joint laxity as measured by the PennHIP distraction index was a major risk factor for developing OA. These results have been confirmed in multiple studies [11] [27] [28] [29] [30]. The University of Pennsylvania Hip Improvement Program (PennHIP) was established that same year, 1993. The DI [6] [31] [27] [28], is a major component of PennHIP and it directly measures the relative amount of laxity present in a canine hip joint as a ratio of two lengths [4] [6], thus making it unitless. It is interpretable, however, as the proportion of femoral head radius that the center of the femoral head moved outward, away from the acetabulum when adequate distractive force was applied [6] while the dog was deeply sedated and dorsally recumbent. A DI value of 0.50, for example, means that the femoral head is 50% out of joint or luxated. The PennHIP method when performed by trained practitioners with hip images read by skilled PennHIP scrutineers, yields DI measurements that are highly repeatable, both within and between examiners and within dog with age [6] [16] [32]. The DI repeatability from 4–6 months, 4–12 months, and 4 to 24 months of age is high (r_i_ = 0.87, 0.82, and 0.85 respectively) [6] so dogs can be evaluated as early as 4 months of age as opposed to one or two years of age for HEV scoring.

The ideal or target phenotype for a DI assessment is a value at or below 0.30 [6] [27]. Dogs with a DI value at or below this threshold have almost no risk of developing OA at any age. It may never be possible for all dogs to possess DI values at or below 0.30, but for dogs with values above 0.30, the closer they are to this threshold value, the lower is their risk of developing OA and the later in life that OA will manifest radiographically [11]. Breeding programs that focus on reducing phenotypic variation in DI below a mean of 0.30 will produce fewer dogs at risk of developing OA of CHD in their lifetimes [15].

### Genetic control programs designed to eliminate CHD

For over 60 years, hip quality assessment schemes based on a qualitative score derived from a HEV radiogrpah have been widely used for characterizing hip quality in dogs [4] [18] [25] [33] [34]. Furthermore, dog breeders have trusted the information provided by these schemes as being actionable information for identifying the best young dogs to keep for breeding [4] [19] [35]. Even though these schemes have been widely used for decades, it remains unproven that using a HEV-based score as the phenotypic selection criterion to identify young dogs to be kept for breeding will actually lower the risk of producing OA susceptible puppies in future generations.

To establish this proof and for a genetic screening system to be effective, three requirements must be satisfied [12] [16]. First, the screening metric or score should be biologically related to the disease phenotype, which is osteoarthritis in this study. Dogs that receive a score that classifies them as being at low risk prior to being selected for breeding should not develop OA later in life. Second, the phenotype used to define the selection criterion must be heritable. This simply means that some significant portion of observed variation in the phenotype is under genetic control. Third, and perhaps most important, the scheme for identifying young breeders must apply selection pressure, which is quantified by the selection differential [36]. The selection differential measures the amount by which the average phenotypic value of parents differs from the average phenotypic value of the population from which the new parents were chosen [36]. When all candidates available for selection to become parents have the same phenotype, the selection differential is zero. When the selection differential is zero, it does not matter how highly heritable the phenotype might have been in earlier generations, there can be no more genetic change because the phenotypes of all prospective parents are identical [36]. These three requirements for producing genetic change in a population are briefly described here because they bring into sharp focus the difference between the relatively small amount of genetic improvement realized by dog breeders using HEV-based scoring schemes [24] [35] [37] and the results reported herein for a similar measure of hip quality.

The Seeing Eye, Inc. (TSE) produces purpose-bred dogs destined for work as guides for blind people. Breeding goals focus on: (a.) improved hip quality, (b.) an increasing ability to be trained for working as guides, and (c.) a reduction in the incidence of all inherited health disorders. As implemented from 1980 through the early 2000’s, TSE’s breeding plan for improving hip quality met requirements 1 and 2, described above, but in later generations, it could no longer meet requirement 3. It is the purpose here to report the results obtained by TSE over 35 years of breeder selection for improved hip quality based on using estimated breeding values (EBVs) to describe germplasm available for selection as replacement breeders.

### Materials and methods

#### Breeding population description

TSE, founded in 1929, began breeding purpose-bred GSDs for work as guides in the 1940’s. The puppies that are the basis of this report were produced by a breeding population descended from foundation breeding stock that were slowly acquired from private dog breeders throughout the U.S. beginning in the early 1970s and continuing through the mid-1980’s. Most replacement breeders, however, were chosen from among puppies born into the program. In later years, a few outside breeding stock were acquired to help maintain genetic diversity, but most of these outside breeders had no permanent impact on the breeding population because none of their offspring were saved for breeding.

Dogs of 3 breeds—German Shepherd Dogs (GSD), Labrador Retrievers (LR), and Golden Retrievers (GR)—were routinely produced, along with some LR x GR crossbred dogs. The hip quality results reported here were obtained only from the purebred dogs, with all hip quality scores on crossbred dogs being excluded from this report. The number of puppies born per year varied from as few as 425 to as many as 680, with about 35% of newborns being purebred GSD’s, 35% purebred LR’s, and the remaining 30% being split approximately equally between purebred GR’s and LR x GR crossbreds. A detailed genetic structure analysis of TSE’s breeding colony through 2002 was previously reported [38].

All breeding bitches and studs were housed in a TSE-owned facility whose entire purpose is the daily maintenance and management of breeding, whelping, and provision of proper veterinary and emotional care of all dogs in residence. Puppies remained with their mother in the breeding center until weaning at about 6 weeks of age. By their 8’th week, each puppy would have been transported to a volunteer puppy raiser’s home, where they were raised and cared for with regular TSE supervision until the puppies reached 14–18 months of age. At 14–18 months of age, dogs return to TSE for extensive medical evaluation, prior to beginning a 4-month or longer training period, where they learn to work as guides for blind people.

In 1978, TSE began obtaining an HEV-based hip quality score (HES) using a 9-point ordinal scale [39] that ordered hip quality from worst (HES = 1) to best (HES = 9), with scores 7 and 8 being similar in meaning to OFA scores of Good and Excellent, respectively. HES values of 1, 2, or 3 were interpreted to mean the HEV image showed evidence of CHD with additional evidence of OA also known as degenerative joint disease (DJD). A HES of 4 meant there was evidence of CHD in the form of subluxation, but no evidence of OA. All HES values were provided by an ACVR-Board Certified radiologist (co-author DNB). Over time, it became clear that score 1 was rarely used and score 9 was never used, so the *de facto* scale actually became a 7-point scale, very similar to the OFA 7-point scale, but with higher quality scores increasing in number, rather than decreasing, as with OFA scores. The final HES value assigned to a dog was determined by the quality score assigned to the worse hip.

As each HES was obtained, it was entered into electronically maintained records, thus enabling the calculation of EBVs beginning in the early 1980’s. These early-day EBVs were calculated by solving a system of up to four selection index equations [36] that took into account the individual dog’s own record, the records of all the dog’s paternal and maternal half-sibs, and all progeny records, if the dog was a parent. To control for possible scoring changes over time, a dog’s own score was expressed as a deviation from the contemporary group mean. A contemporary group was arbitrarily defined as all dogs of the same breed passing through the production system in each unique calendar quarter. For most contemporary groups, this yielded 30–50 dogs per group. These deviated scores became the adjusted phenotypic records actually used in the EBV calculations, and this approach to calculating EBVs based on HES was used until 1995.

Routine PennHIP distraction radiography was initiated in 1990. From 1990–1994, all DI values were retained by the University of Pennsylvania as part of their on-going research project testing validity of the PennHIP method for assessing hip quality. Beginning in 1995, DI measurements on TSE-bred dogs were stored alongside HES in the TSE database. Prior to mid-1995, all hip quality selection decisions were entirely based on EBVs for the HES. From 1995 through 2005 (approximately), hip quality selection decisions were based on EBVs calculated for both HES and DI values. Since 2005, only the DI EBVs and observed phenotypes have been used to decide, with respect to hip quality, which dogs will be allowed to reproduce.

In 1995, TSE upgraded the electronic record keeping system and began calculating EBVs using MTDFREML [40], This software enabled simultaneous fitting of thousands of equations on multiple traits, obtaining in the process both estimates of variance components for individual traits and estimates of covariance between related traits. The software also calculated EBVs by taking into account all pedigree relationships and important fixed effects for each trait. Since the release of MTDFREML roughly coincided with the availability of PennHIP DI scores on dogs evaluated from 1990–1994, PennHIP became another trait for which EBVs were calculated.

### Inclusion criteria and radiographic methods

Records on purebred GSD, GR, and LR puppies born at TSE from 1976 through fiscal year 2013 were included in the study if they had been evaluated for hip quality. The study dataset also included all zero generation parents if they, themselves, had been evaluated for hip quality. Hip quality radiographs performed at 12–18 months of age were obtained as part of routine physical examinations performed to medically qualify each dog for working as a guide. Because all hip quality data were obtained during routine medical screening, no Animal Care and Use committee approval was sought or required. Radiographic images of each dog were obtained under deep sedation, using varied protocols that were chosen based on the specific dog and appropriate pharmaceuticals.

For the HEV radiograph, dogs were sedated and placed in a supine position. The hips were fully extended with femora pronated such that the patellae were superimposed centrally over the trochlear grooves. Each ventrodorsal HEV radiograph was collimated to include the caudal lumbar spine and the stifles. PennHIP radiographic evaluation consists of three radiographic views: the same hip-extended view, plus a compression, and distraction radiographic view with legs in a neutral position [6]. Hip radiographs were obtained by TSE veterinarians or veterinary technicians, all of whom were trained and certified by PennHIP according to their standard certification requirements [12]. DI measurements were obtained using PennHIP standard protocols [6] [12].

### Pedigree definition and estimation of genetic parameters

Pedigree relationships were extracted from electronic records maintained by TSE. All available ancestral records for each litter were used to calculate inbreeding coefficients using standard techniques [41]. To track genetic change over generations of selection and in the presence of overlapping and partial generations, an approximate generation coefficient was calculated for each litter by adding 1.0 to the average generation coefficients of parents [42]. Using this technique enabled accommodating overlapping generations, and by rounding each dog’s generation coefficient into whole generation classes, it provided a convenient way to summarize average change in phenotypic values in response to selection.

For each breed, a multi-trait modeling approach was used to obtain estimates of genetic and residual variance for each trait, along with estimates of genetic and residual covariances between them. To account for non-genetic factors that could also have contributed to variation observed in these two measures of hip phenotype, each record was assigned to both a contemporary group, as described above, and to a generation class.

The statistical model explaining variation in DI included separate terms for gender and generation class of the dog, a covariate adjusting for differences in level of inbreeding, and a random additive genetic effect (breeding value) for each dog. Preliminary models were used to examine the importance of age at radiographic evaluation as a covariate, along with maternal and litter effects as additional random effects. None of these potential sources of variation was significant, so each was excluded from the final model.

For genetic analyses, HES was considered to be a linear measure of hip quality as a continuous variable with values ranging from 1 to 8. The statistical model explaining variation in the HES included terms for gender, contemporary group, and generation class of the dog, a covariate adjusting for age (in days) when the hip-extended radiograph was taken, a covariate adjusting for a possible effect of inbreeding, and a random additive genetic effect (breeding value) for each dog. As with the model for DI, preliminary models for HES considered the possibility that maternal and litter effects could have been important, but they were not, so were excluded from the final model. For both hip quality phenotypes, pedigree relationships among dogs were included in the models through the inverse of the numerator relationship matrix, thus accounting for all degrees of relationships among pairs of dogs.

Estimates of genetic parameters were obtained by two different approaches: (1.) restricted maximum likelihood as implemented by MTDFREML [40], and (2.) a Bayesian method implemented by *bayz 2*.*04* [43]. For MTDFREML, the final convergence criterion was set at 1.0E-9. For the Bayesian analyses, 500,000 iterations were done. The first 50,000 rounds were discarded as burn-in, followed by thinning the remaining rounds at the rate of 1 kept per each 500 in the MCMC chain.

Analyses of variance testing statistical significance of explanatory variables on the dependent variable were obtained using the "aov" procedure as implemented in "R" [44]. Other R modules used in completing this study included "epiR" [45], "HMisc" [46], "RMySQL" [47], “epibasix” [48] and “ggplot2” [49]. The statistical significance of changes in the distribution of HES observed across generation classes was assessed using the non-parametric Jonckheere-Terpstra [50] statistic as implemented by the “clinfun” [51] module in “R”.

## Results

### Animals

The final dataset for HES included 12,496 dogs: 5,201 GSD, 4,987 LR, and 2,308 GR. Average age (+/- SD) for those dogs at hip evaluation was: 461d (+/- 92d) for GSD; 475d (+/- 96d) for LR; and 503d (+/- 160d) for GR. Overall, the male:female sex ratio was 1.048, while within breed, the sex ratios were: 0.983 in GSD, 1.092 in LR, and 1.110 in GR.

The final dataset for DI included 9,548 dogs: 3,782 GSD, 3,662 LR, and 2,104 GR. Mean ages (+/- SD) of those dogs at hip evaluation were: 486d (+/- 88d) for GSD, 500d (+/- 91d) for LR, and 508d (+/- 150d) for GR. Across breeds, the male:female ratio was 1.058. Within breed, the sex ratios were: 0.985 in GSD, 1.108 in LR, and 1.108 in GR. The 2,948 dogs without a DI measurement were, for the most part, dogs that passed through the system prior to the beginning of distraction radiography in 1991. Fewer than 100 dogs born prior to 1990 had DI values recorded, and when a DI value was recorded for these older dogs, it was usually obtained as part of the routine physical given to retiring breeders.

To account for all meaningful pedigree relationships within a breed, additional pedigree records were added for ancestors on whom no hip quality measurements had been recorded. This resulted in the addition of 219 GSD, 424 LR, and 283 GR records to the respective pedigree files.

### Measures of hip quality: Hip-extended score (HES)

HES values observed in three breeds ranged from 1 to 8, with poorer quality hips receiving lower scores. To ease the translation of results reported herein to other HEV-based hip quality studies reported in the literature, the 8 classes used for genetic analyses have been reduced to 7 classes for summary purposes. The terms “Excellent”, “Good”, and “Fair” are equivalent to HES values of 8, 7, and 6 respectively. “Borderline” is equivalent to HES = 5, while “Mild CHD” and “Moderate CHD” are equivalent to HES values of 4 and 3, respectively. “Severe CHD” represents the HES values of 2 and 1, but it must be noted that HES = 1 was used in less than 1% of cases for each breed. Combining HEV score classes 1 and 2 results in negligible loss of information, so this collapsed scale is called HESC in the remainder of this report.

The first dogs evaluated by HES were born in 1976, although it was in 1980 before all TSE-bred dogs were routinely evaluated by this standardized protocol. By 1981, all prospective parents would have been evaluated using HEV radiography. By breed, the percent of dogs classified into each of 7 HESC classes is given in Table 1 for each generation class. The small numbers of generation 0 dogs shown for each breed in Table 1 are the few founders on which HESC was obtained. In each breed, the puppies born into generation 1 were produced by foundation parents, a few of which had HESC values, but most did not.

**Table 1:** Percent of dogs within breed and generation class classified by degree of hip dysplasia, as reflected by the hip-extended score.

Hip-Extended Score Collapsed (HESC) from 8 into 7 Classes										
Generation Class	Severe	Moderate	Mild	Border-line	Fair	Good	Excellent	Total N Observed	Some Degree of CHD	Normal, with no CHD
German Shepherd Dogs										
0	0.0%	0.0%	0.0%	0.0%	4.3%	4.3%	91.3%	23	0.0%	100.0%
1	17.9%	11.0%	19.0%	5.9%	1.8%	10.6%	33.7%	273	53.8%	46.2%
2	10.7%	13.5%	25.7%	4.3%	3.1%	9.3%	33.4%	891	54.1%	45.9%
3	6.5%	9.9%	32.9%	1.2%	0.7%	12.2%	36.6%	434	50.5%	49.5%
4	0.8%	3.4%	18.7%	0.2%	1.9%	25.5%	49.5%	475	23.2%	76.8%
5	0.2%	3.4%	13.8%	0.4%	2.8%	27.7%	51.7%	470	17.9%	82.1%
6	0.2%	1.9%	5.2%	0.2%	1.4%	12.6%	78.5%	517	7.5%	92.5%
7	0.2%	1.0%	1.5%	0.0%	0.2%	4.4%	92.7%	592	2.7%	97.3%
8	0.0%	0.3%	2.9%	0.0%	0.4%	3.0%	93.4%	695	3.2%	96.8%
9	0.0%	0.6%	1.7%	0.0%	0.2%	1.5%	96.0%	525	2.3%	97.7%
10	0.0%	0.0%	1.0%	0.0%	1.0%	2.7%	95.2%	294	1.0%	99.0%
11	0.0%	0.0%	0.0%	0.0%	0.0%	0.0%	100.0%	12	0.0%	100.0%
Total								5201		
Labrador Retrievers										
0	0.0%	5.6%	0.0%	0.0%	0.0%	11.1%	83.3%	18	5.6%	94.4%
1	7.7%	7.4%	16.1%	9.3%	1.5%	3.4%	54.5%	323	40.6%	59.4%
2	3.2%	4.3%	14.8%	2.2%	2.2%	4.6%	68.7%	648	24.5%	75.5%
3	1.7%	5.1%	14.2%	0.3%	0.9%	10.0%	67.8%	633	21.3%	78.7%
4	0.4%	2.2%	7.5%	0.0%	1.4%	12.4%	76.3%	510	10.0%	90.0%
5	0.2%	1.6%	4.2%	0.0%	4.2%	17.7%	72.1%	548	6.0%	94.0%
6	0.2%	0.9%	1.3%	0.0%	0.2%	10.2%	87.3%	550	2.4%	97.6%
7	0.1%	0.6%	0.4%	0.0%	0.0%	11.2%	87.7%	717	1.1%	98.9%
8	0.0%	0.0%	0.2%	0.0%	0.0%	5.5%	94.3%	528	0.2%	99.8%
9	0.0%	0.3%	0.3%	0.0%	0.0%	1.3%	98.2%	396	0.5%	99.5%
10	0.0%	0.0%	0.0%	0.0%	0.0%	0.0%	100.0%	116	0.0%	100.0%
Total								4987		
Golden Retrievers										
0	0.0%	12.5%	0.0%	0.0%	12.5%	12.5%	62.5%	8	12.5%	87.5%
1	2.0%	13.7%	17.6%	0.0%	3.9%	19.6%	43.1%	51	33.3%	66.7%
2	6.3%	6.3%	20.3%	0.0%	0.0%	6.3%	60.8%	79	32.9%	67.1%
3	1.5%	6.1%	15.2%	0.0%	0.8%	16.7%	59.8%	132	22.7%	77.3%
4	0.2%	6.2%	14.0%	0.0%	3.8%	17.8%	57.9%	449	20.5%	79.5%
5	0.2%	4.2%	10.8%	0.2%	2.1%	19.3%	63.3%	529	15.3%	84.7%
6	0.0%	5.5%	6.2%	0.0%	0.4%	18.3%	69.6%	471	11.7%	88.3%
7	0.0%	1.5%	3.6%	0.0%	0.2%	13.5%	81.2%	473	5.1%	94.9%
8	0.0%	0.0%	0.9%	0.0%	0.0%	12.1%	87.1%	116	0.9%	99.1%
Total								2308		

#### German Shepherd Dogs: HESC results (Table 1, Fig 1)

**Fig 1:**
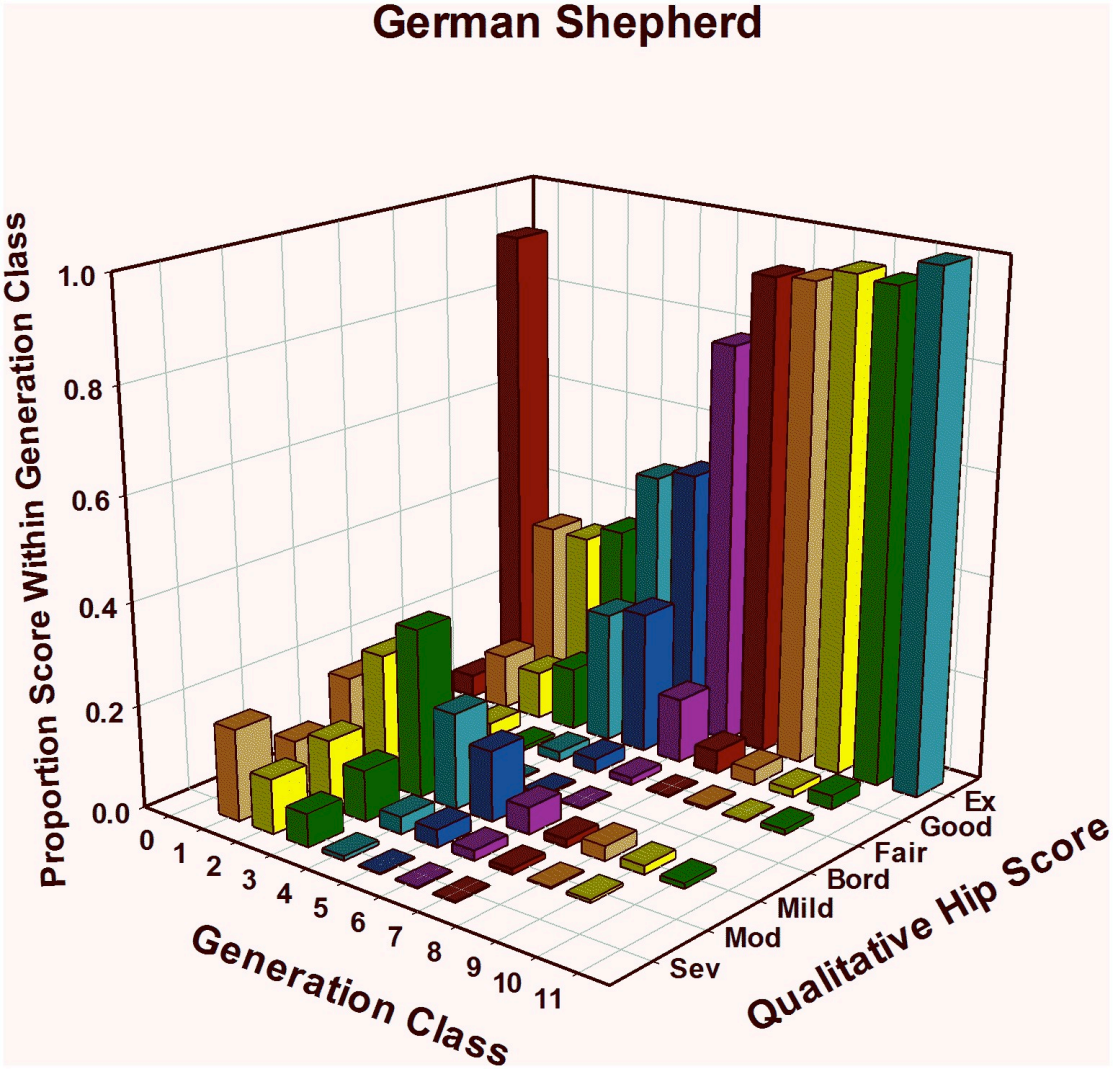
Genetic change in hip extended score across 11 generations of selection in German Shepherd Dogs.

Among 273 generation 1 GSD puppies evaluated by HESC, 46% were classified as having normal hips, while 54% had hips showing signs of CHD from borderline to severe (Fig 1). The production of 891 generation 2 puppies produced little macro-level change, with 46% classified as normal and 54% as showing radiographic signs of CHD, but there was some evidence of a shift beginning in the worst HESC classes. Among those with CHD, fewer were classified as severe while more were classified as mild than in generation 1. This progression—with fewer severe CHD dogs and more mild CHD dogs—was repeated in generations 2 and 3, but there was only a small increase from the mid- to the high-40’s in percent of dogs classified as normal. That trend accelerated beginning in generation 4, where genetic change, measured by the percent of dogs classified as fair, good, or excellent, jumped from below 50% to above 75% in one generation. By the end of the 7th generation, over 97% of offspring were classified as having normal hips. In generations 9, 10, and 11, not only were most dogs classified as normal, most were classified as Excellent: 96% (504 of 525) in the 9^th^ generation; 95% (280 of 294) in the 10^th^ generation; and 100% (12 of 12) in the 11^th^ generation. It must be noted that, at the end of the study period, dogs in all three generation classes 9, 10, and 11 were still being produced, so the reported percentages are preliminary for those classes. Among 5,201 GSDs evaluated using the HESC, the null hypothesis that the distribution of observed values among generation classes arose from one common underlying distribution was rejected (JT = 1538, P<0.001) in favor of the alternate hypothesis that the HESC distribution changed as generation classes increased. Clearly, HESC shifted toward the Excellent end of the scale as generations of selection operated to change the HEV phenotype (Fig 1). By the end of the study period, 95.8% of 831 dogs evaluated in the combined generation classes 9, 10, and 11 had HESC Excellent hips, leaving only 35 (4.2%) dogs with less than Excellent ratings. Of those 35, 16 were classified as Good, and 4 as Fair, while 12 showed signs of mild and 3 of moderate CHD.

#### Labrador Retrievers: HESC results, (Table 1, Fig 2)

**Fig 2:**
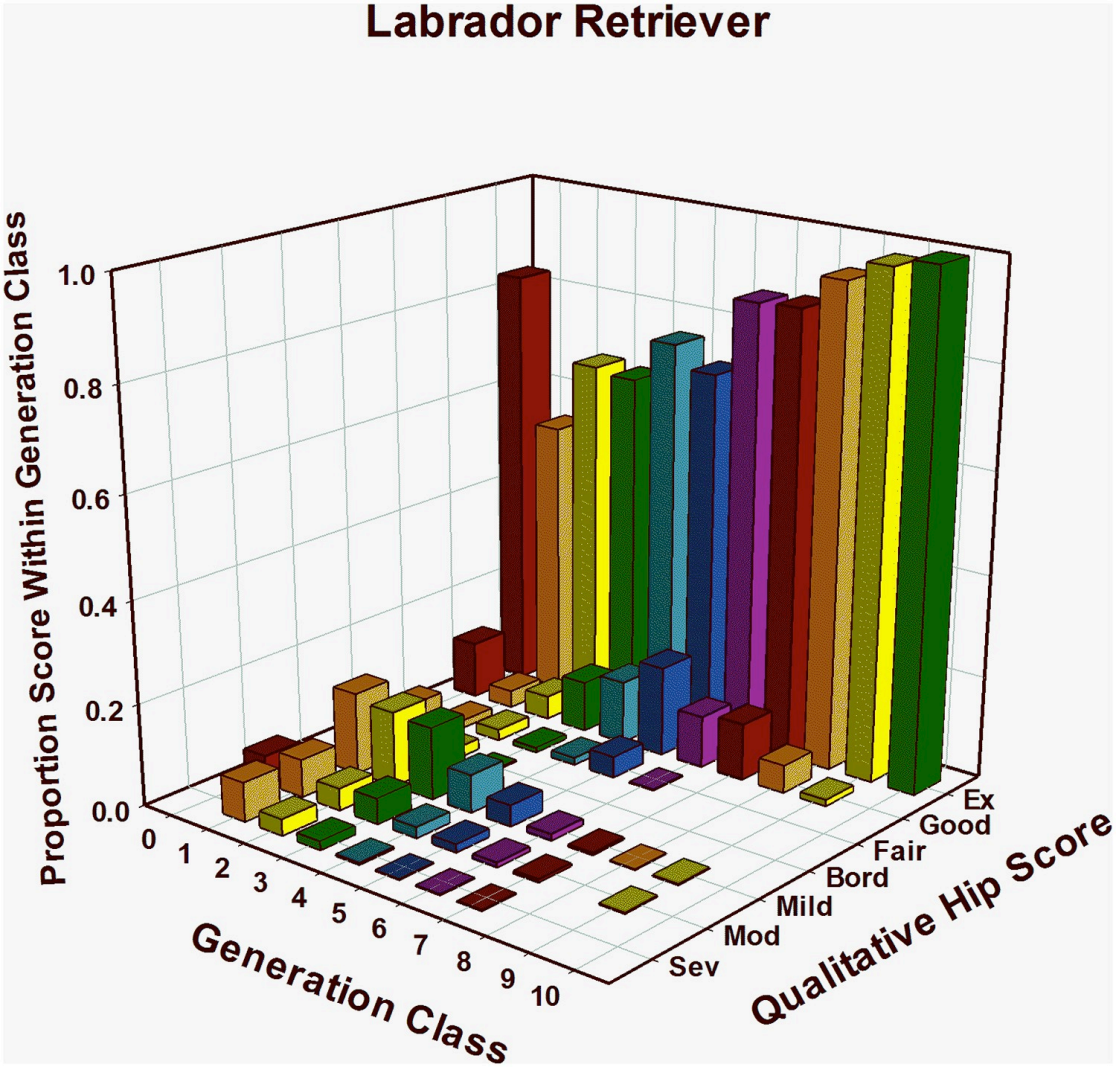
Genetic change in hip extended score across 10 generations of selection in Labrador Retrievers.

Among 323 LR puppies born in generation 1, 59% were classified as having HESC normal hips (HESC = Fair, Good, or Excellent), while 41% were classified as having some degree of CHD, from Borderline to Severe (Fig 2). In generation 2, there was a dramatic shift toward normal, with almost 76% receiving Fair (2.2%), Good (4.6%), or Excellent (68.7%) scores, from among 648 dogs evaluated (Table 1). Generation class 3 produced a distribution across HESC values similar to those observed in generation 2. Beyond generation 3, however, there was a steady shift toward an increasing percentage of dogs classified as Excellent and a decreasing percentage classified as having Severe or Moderate CHD (Table 1). Among 396 dogs in generation 9 evaluated using HESC, over 98% (389 dogs) were classified as HESC Excellent. In generation 10, every dog evaluated (100% of 116 dogs) was classified as HESC Excellent (Fig 2). Among 4,987 LRs classified by HESC, the distribution of HEV phenotypes steadily and significantly (JT = 1256, P<0.001) shifted toward the Excellent phenotype as generation classes moved forward in time (Table 1, Fig 2.). Over 1,000 dogs were evaluated in the last 3 generation classes. Among them, 96% (1,003 of 1,040) were classified as having Excellent hips. Another 3% (34 of 1,040) had Good hips, leaving only 3 dogs that showed radiographic signs of CHD.

#### Golden Retrievers: HESC results, (Table 1, Fig 3)

**Fig 3:**
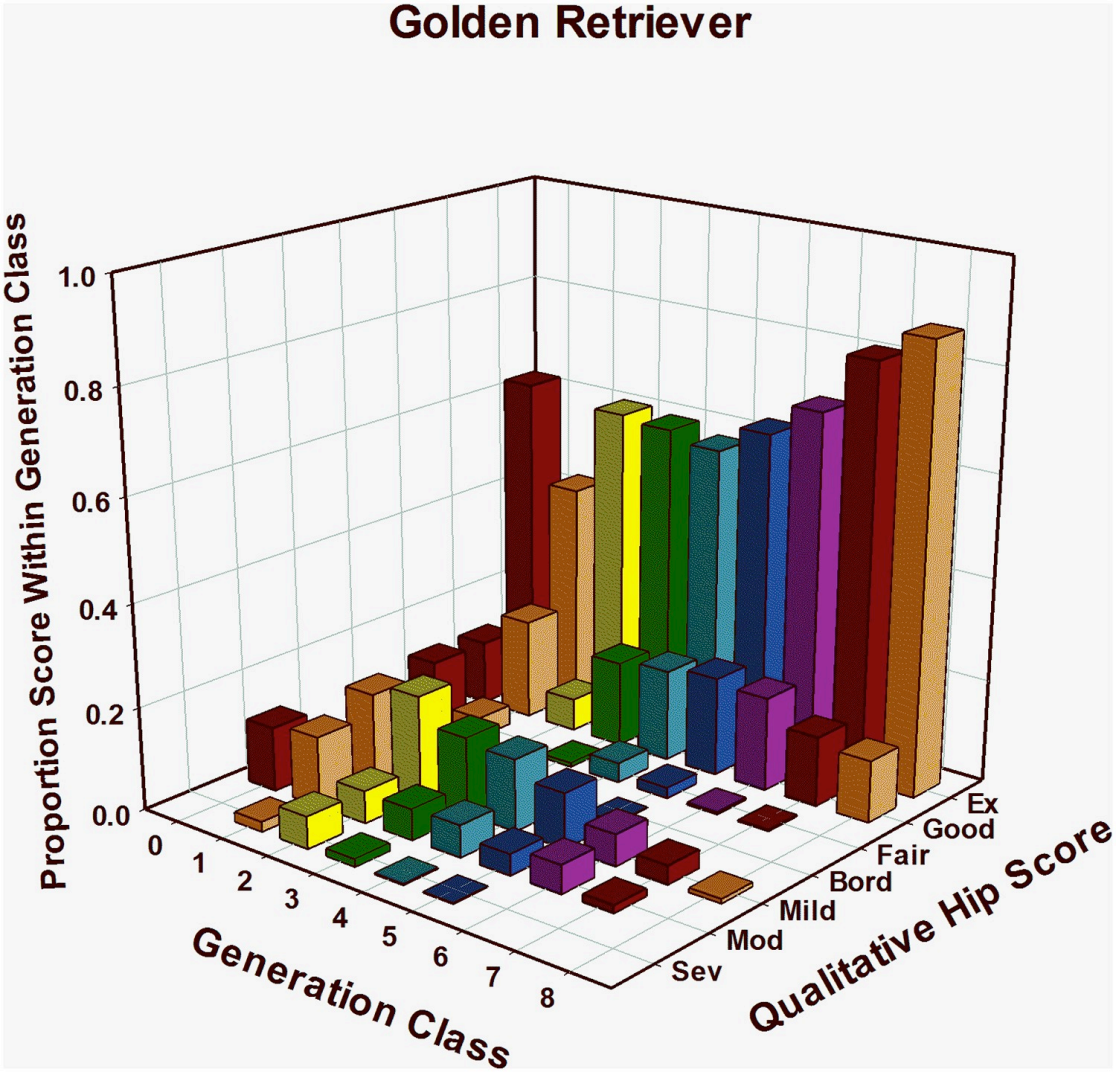
Genetic change in hip extended score across 8 generations of selection in Golden Retrievers.

GRs, which were only produced in significant numbers beginning in the early 1990’s, have been a minor breed in TSEs breeding program. Even so, hip quality using HES was evaluated on over 2,300 GRs. Among 51 1st generation offspring, 67% were classified as having normal hips, leaving 33% that showed evidence of CHD from Borderline to Severe. In generation 2, 79 offspring born had an HESC distribution very similar to those in generation 1, but noticeable improvement appeared among 3^rd^ generation offspring. Over 77% of 132 dogs evaluated in generation 3 were classified as having normal hips, and by the end of the 7^th^ generation, almost 95% of dogs had normal hips at about 16 months of age. This trend continued to the end of the study period, where all but 1 of 116 pups evaluated in the 8^th^ generation class received a Good (12.1%), or Excellent (87.1%) rating (Table 1, Fig 3). By the end of the study period, 2,308 GRs had been evaluated for the HEV phenotype. As genetic selection for improved hip quality moved through successive generations, the distribution of HESC significantly (JT = 1028, P<0.034) shifted toward the Excellent end of the phenotypic scale (Fig 3).

#### Measures of hip quality: PennHIP distraction index (DI)

DI values recorded on 9,548 dogs across 3 breeds ranged from 0.13 to 1.14, with a mean of 0.395 (SD = 0.118). The theoretically possible range is from 0.05 to a value greater than 1, with looser, poorer-quality hips receiving higher scores [6]. For the entire dataset, quartiles were: Q25 = 0.32, Q50 = 0.37, and Q75 = 0.44, but the within breed values (Table 2.) are much more informative because breed differences are highly significant (F_2,9200_ = 930.1, P<0.001).

**Table 2:** Summary statistics describing distraction index values observed among three dog breeds, stratified by generation class.

Generation Class	Min	1st Quartile	Median	Mean	3rd Quartile	Max	No. Obs.	Standard Error of Mean
German Shepherd Dogs								
0	0.25	0.28	0.30	0.33	0.36	0.43	3	0.050
1	0.19	0.30	0.42	0.42	0.49	0.64	12	0.025
2	0.15	0.31	0.35	0.39	0.46	0.69	66	0.011
3	0.14	0.29	0.35	0.37	0.43	0.75	213	0.006
4	0.13	0.29	0.33	0.36	0.40	0.78	422	0.004
5	0.20	0.31	0.36	0.38	0.43	0.79	457	0.004
6	0.19	0.31	0.35	0.37	0.41	0.77	512	0.004
7	0.20	0.31	0.35	0.36	0.40	0.70	587	0.004
8	0.18	0.32	0.36	0.37	0.41	0.80	684	0.003
9	0.22	0.32	0.37	0.37	0.41	0.78	521	0.004
10	0.19	0.29	0.33	0.33	0.36	0.63	293	0.005
11	0.26	0.28	0.30	0.32	0.36	0.40	12	0.025
Overall	0.13	0.31	0.35	0.365	0.41	0.80	3782	0.001
Labrador Retrievers								
0	0.22	0.34	0.40	0.40	0.47	0.52	7	0.043
1	0.24	0.28	0.33	0.33	0.38	0.43	3	0.066
2	0.21	0.32	0.36	0.38	0.44	0.75	39	0.018
3	0.14	0.32	0.38	0.42	0.48	1.14	294	0.007
4	0.17	0.32	0.38	0.42	0.48	0.96	482	0.005
5	0.17	0.32	0.38	0.40	0.45	1.00	546	0.005
6	0.20	0.29	0.35	0.36	0.39	0.83	544	0.005
7	0.17	0.30	0.35	0.36	0.40	0.73	711	0.004
8	0.17	0.31	0.35	0.36	0.40	0.80	525	0.005
9	0.14	0.29	0.32	0.33	0.37	0.81	395	0.006
10	0.19	0.26	0.30	0.30	0.32	0.47	116	0.011
Overall	0.14	0.30	0.35	0.374	0.42	1.14	3662	0.002
Golden Retrievers								
0	0.40	0.42	0.43	0.49	0.54	0.70	7	0.047
1	0.30	0.43	0.52	0.55	0.65	0.91	41	0.020
2	0.40	0.48	0.54	0.58	0.64	0.88	13	0.035
3	0.27	0.42	0.50	0.51	0.60	0.83	106	0.012
4	0.25	0.40	0.48	0.50	0.58	0.91	385	0.006
5	0.24	0.41	0.48	0.50	0.58	0.92	510	0.006
6	0.25	0.39	0.47	0.48	0.56	0.91	458	0.006
7	0.21	0.39	0.46	0.48	0.54	0.91	468	0.006
8	0.19	0.34	0.39	0.40	0.44	0.69	116	0.012
Overall	0.19	0.39	0.46	0.487	0.56	0.92	2104	0.003

#### German Shepherd Dogs: PennHIP DI (Table 2, Fig 4)

Mean DI among 3,782 GSDs was 0.365 (SEM = 0.001, SD = 0.0873). Quartiles among the GSD DI values were: Q25 = 0.31, Q50 = 0.35, Q75 = 0.41, with a minimum observed value of 0.13 and a maximum of 0.80, as shown in Table 2 across 12 generation classes. Few dogs were evaluated for DI in generation classes 0, 1 and 2, simply because the PennHIP methodology was being developed during the years when those generation classes were born, but in generation class 3, 213 GSDs were evaluated. From that point onward, all TSE-bred dogs returning to begin training were evaluated using both DI and HES.

**Fig 4:**
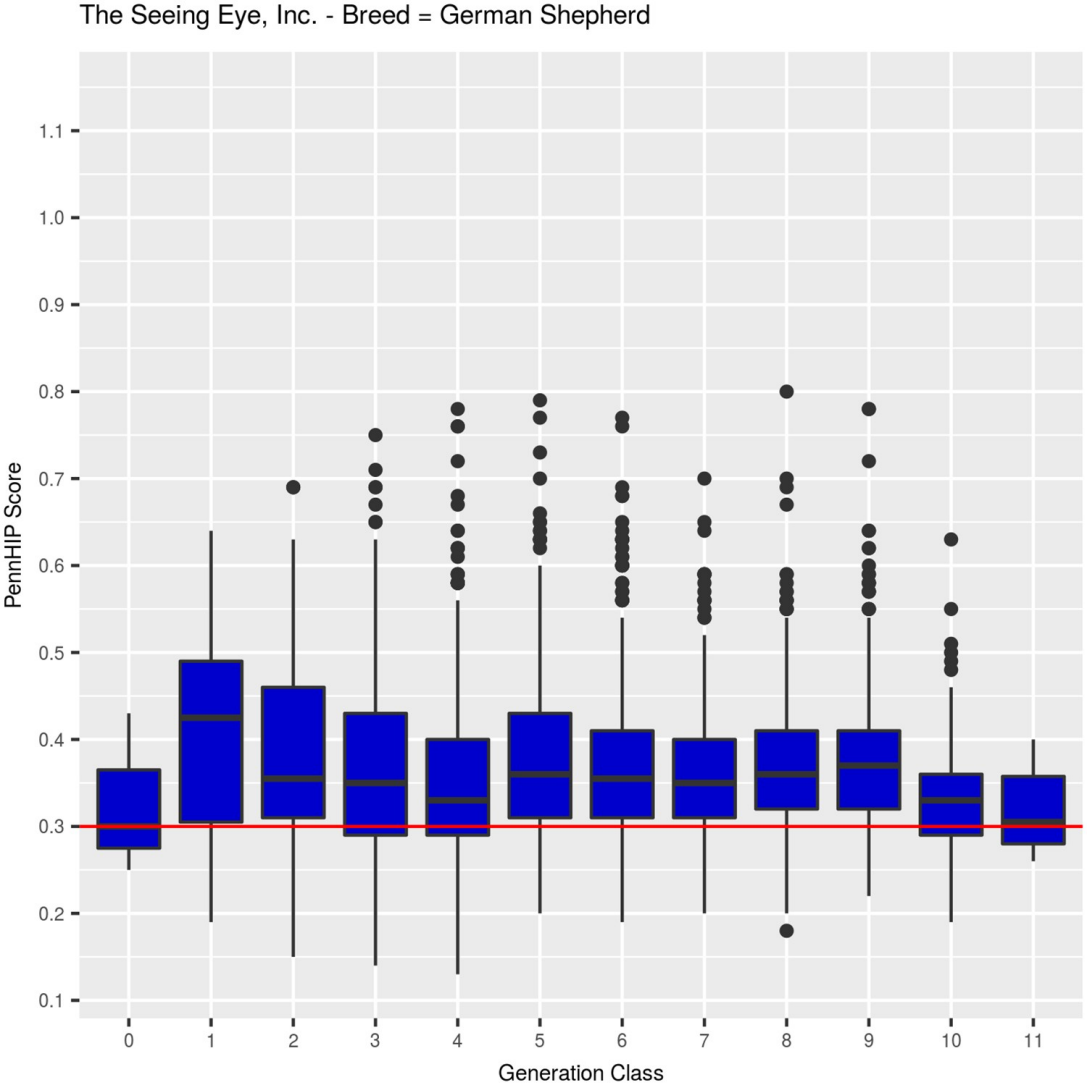
PennHIP distraction index values summarized by generation class for German Shepherd Dogs.

Across 12 generation classes, mean DI values fluctuated around the overall mean of 0.365 (Fig 4), but no discernible change in mean DI values across generation classes was detected (F_1,3778_ = 0.859, P>0.35) when fitted as a covariate. When fitted as a class effect, however, mean DI values across generation classes were significantly different (F_11,3768_ = 9.2, P<0.001), reflecting the higher mean value observed in generation 1 (0.42, N = 12) and the decreasing means observed in generation classes 10 (0.33, N = 293) and 11 (0.32, N = 12) (Fig 4). Between generations 2 and 9, means fluctuated narrowly between 0.36 and 0.39, with no obvious discernible trend.

**Fig 5:**
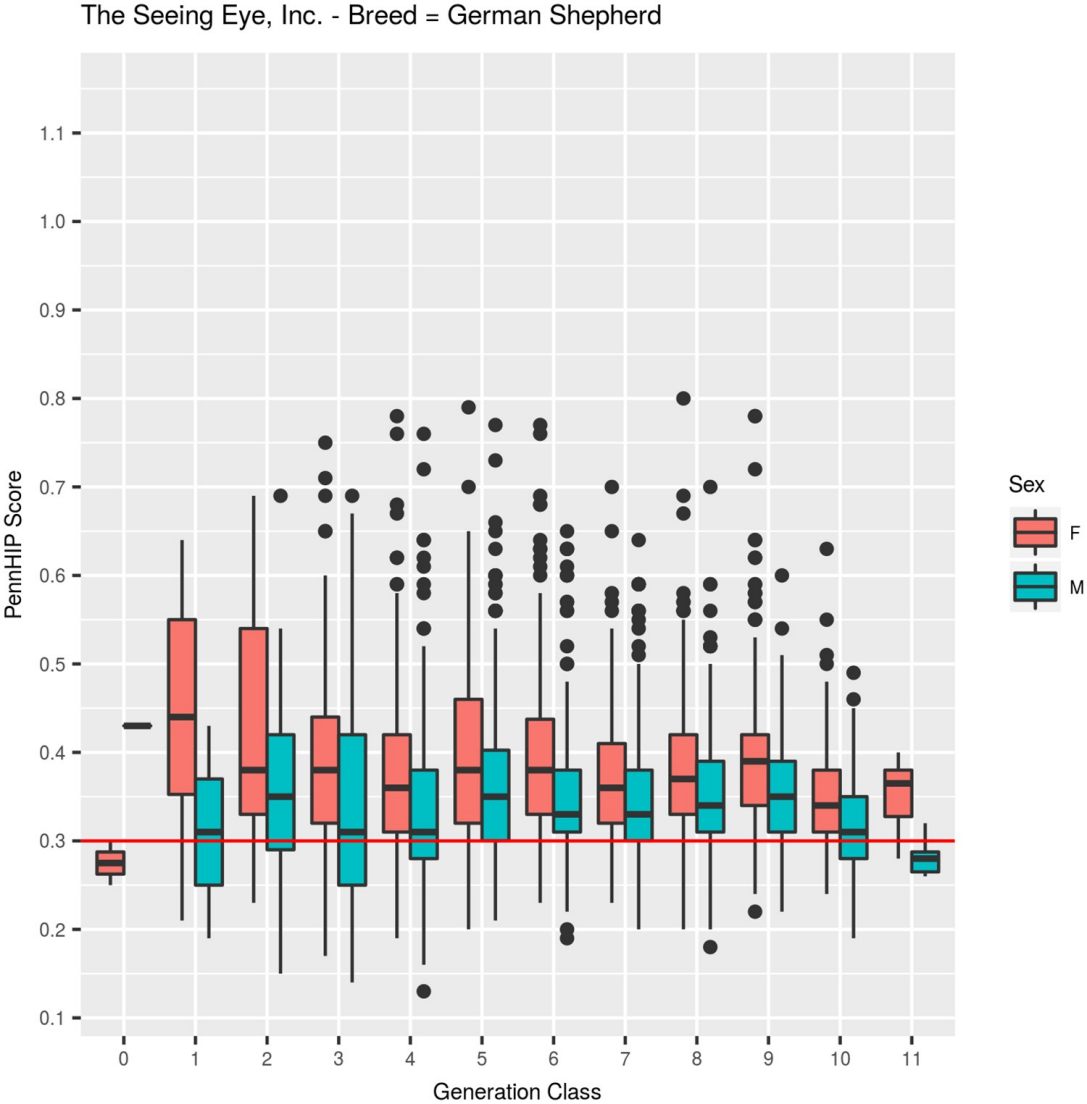
PennHIP distraction index values summarized by sex within generation class for German Shepherd Dogs.

The sex effect was highly significant (F_1,3768_ = 149.1, P<0.001), with males having an average DI value (0.348, N = 1,877) that was 0.034 units below females (0.382, N = 1,905) (Fig 5). Inbreeding coefficients among GSDs averaged 17.4%, but ranged in value from 0 to 37.9%, with 50% of the values falling between 12.8% and 22.9%. For each 1% increase in inbreeding coefficients of GSDs, DI decreased, on average, by 0.001 units (F_1,3778_ = 44.9, P<0.001).

#### Labrador Retrievers: PennHIP DI, (Table 2, Fig 6)

**Fig 6:**
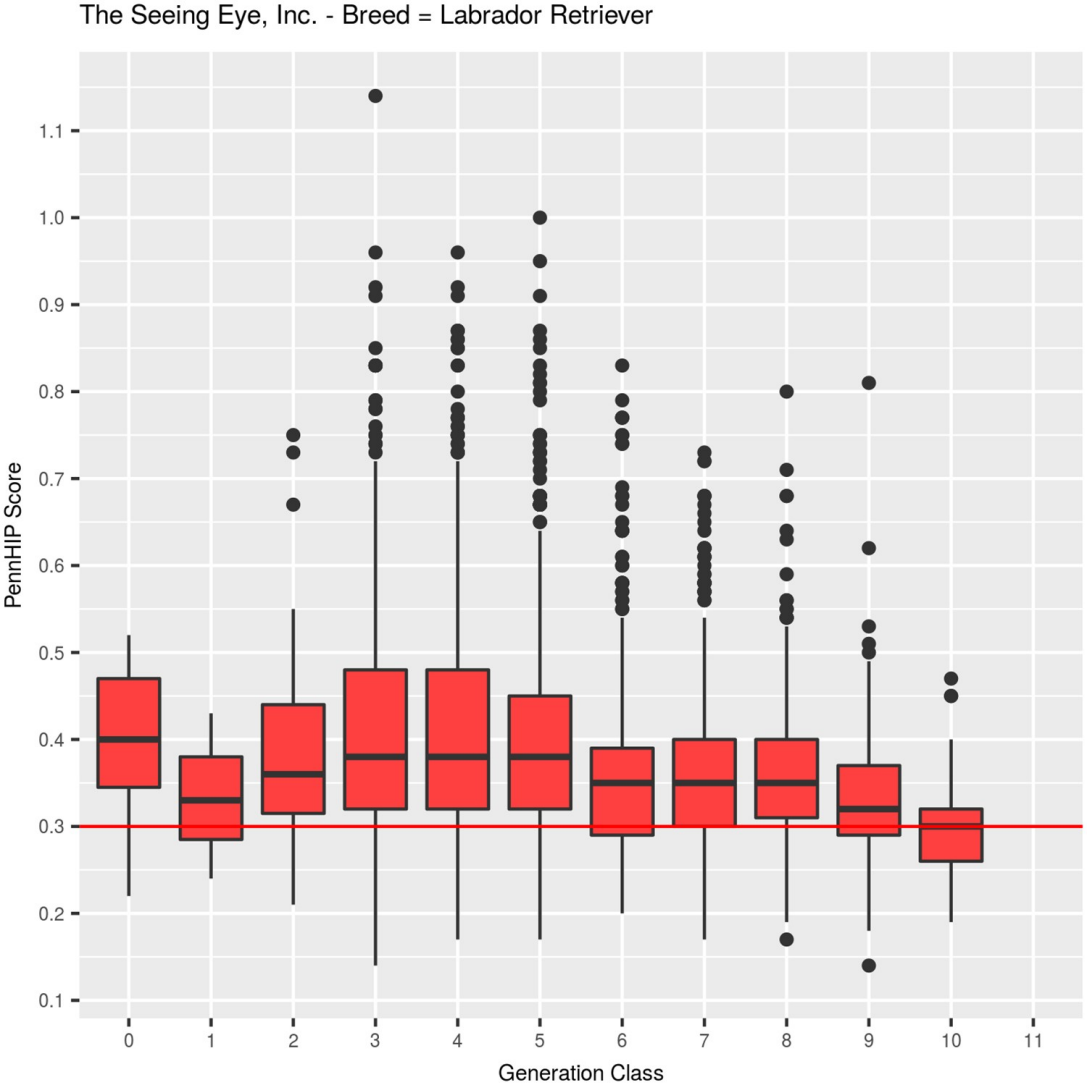
PennHIP distraction index values summarized across 11 generation classes for Labrador Retrievers.

Among 3,662 LRs, mean DI was 0.374 (SEM = 0.002, SD = 0.1142), with values ranging from a minimum of 0.14 to a maximum of 1.14 (Table 2). The observed quartiles were: Q25 = 0.30, Q50 = 0.35, and Q75 = 0.42. DI values were observed across 11 generation classes, but it must be noted that very few dogs were evaluated in generation classes 0 (N = 7), 1 (N = 3), or 2 (N = 39). Recording of DI values in larger numbers began for dogs born in 1990 and later, which means that a full decade of HES-based genetic selection occurred before the first DI values were obtained. Among 294 dogs born in generation class 3, mean DI was 0.42. Over generation classes 4 through 9, mean DI value moved downward in a stair step manner (.42, .42, .40, .36, .36, .36, respectively), with a large change observed between generations 5 and 6, but then no change occurred in generations 6, 7, or 8. That picture changed rather dramatically, however, in generations 9 (0.33, N = 395) and 10 (0.30, N = 116), where DI values declined by 0.06 DI units in two generations (Fig 6). Overall, the decline in generation class means was highly significant, whether fitted as a covariate (F_1,3658_ = 187.26, P<0.001) or as a class effect (F_10,3649_ = 23.76, P<0.001).

**Fig 7:**
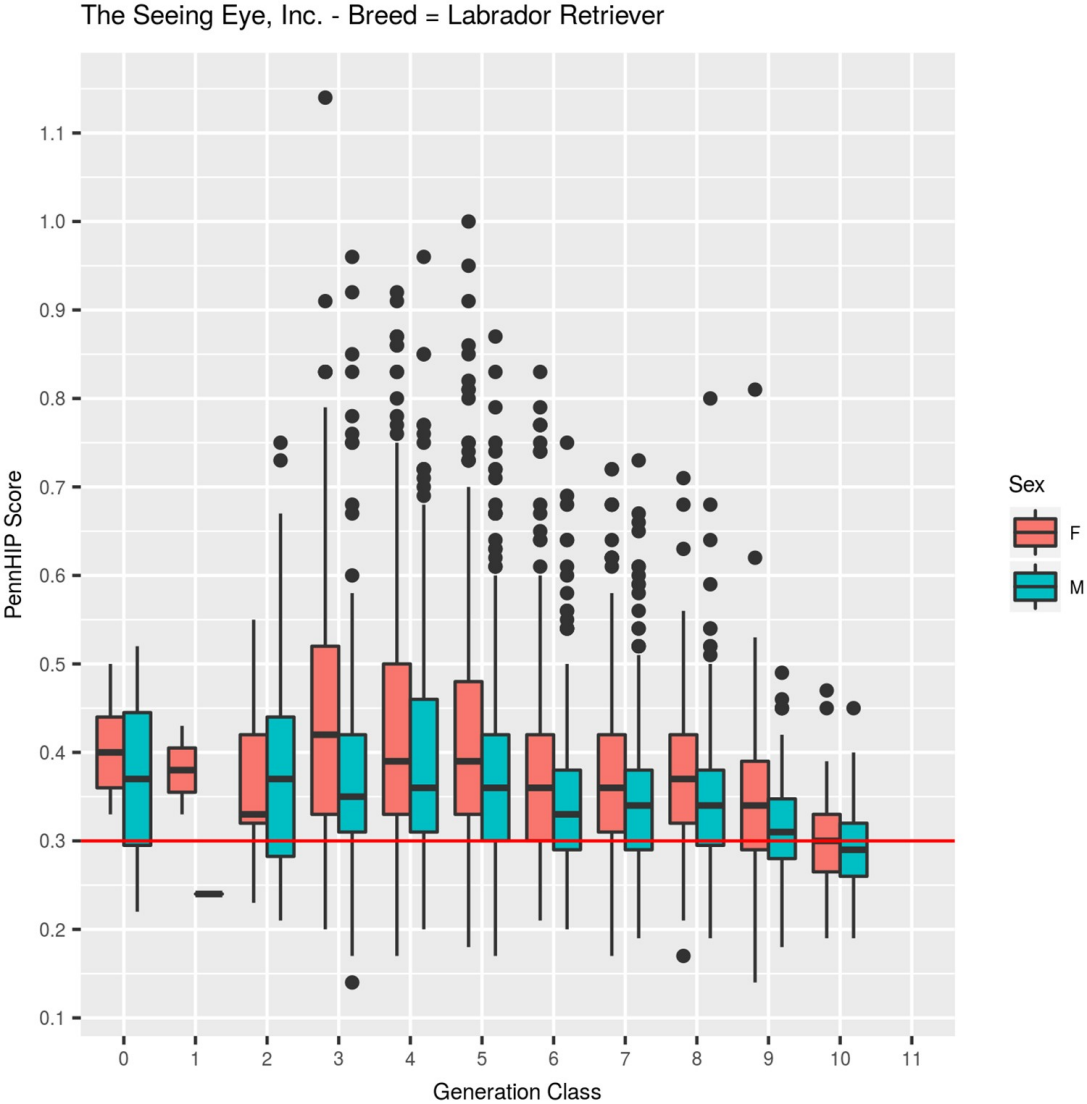
PennHIP distraction index values summarized by sex within generation class for Labrador Retrievers.

The difference in mean DI between males (0.358, N = 1,925) and females (0.393, N = 1,737) was -0.035 units, which was highly significant (F_1,3649_ = 95.1, P<0.001), Fig 7. Inbreeding coefficients in LRs ranged from 0 to 26.5%, with a mean of 10.9%, and quartiles of: Q25 = 7.0%, Q50 = 11.9%, and Q75 = 15.3%. As inbreeding increased by 1%, DI decreased, on average, by 0.001 units, which was a highly significant (F_1, 3649_ = 92.6, P<0.001) decrease.

#### Golden Retriever: PennHIP DI, (Table 2, Fig 8)

**Fig 8:**
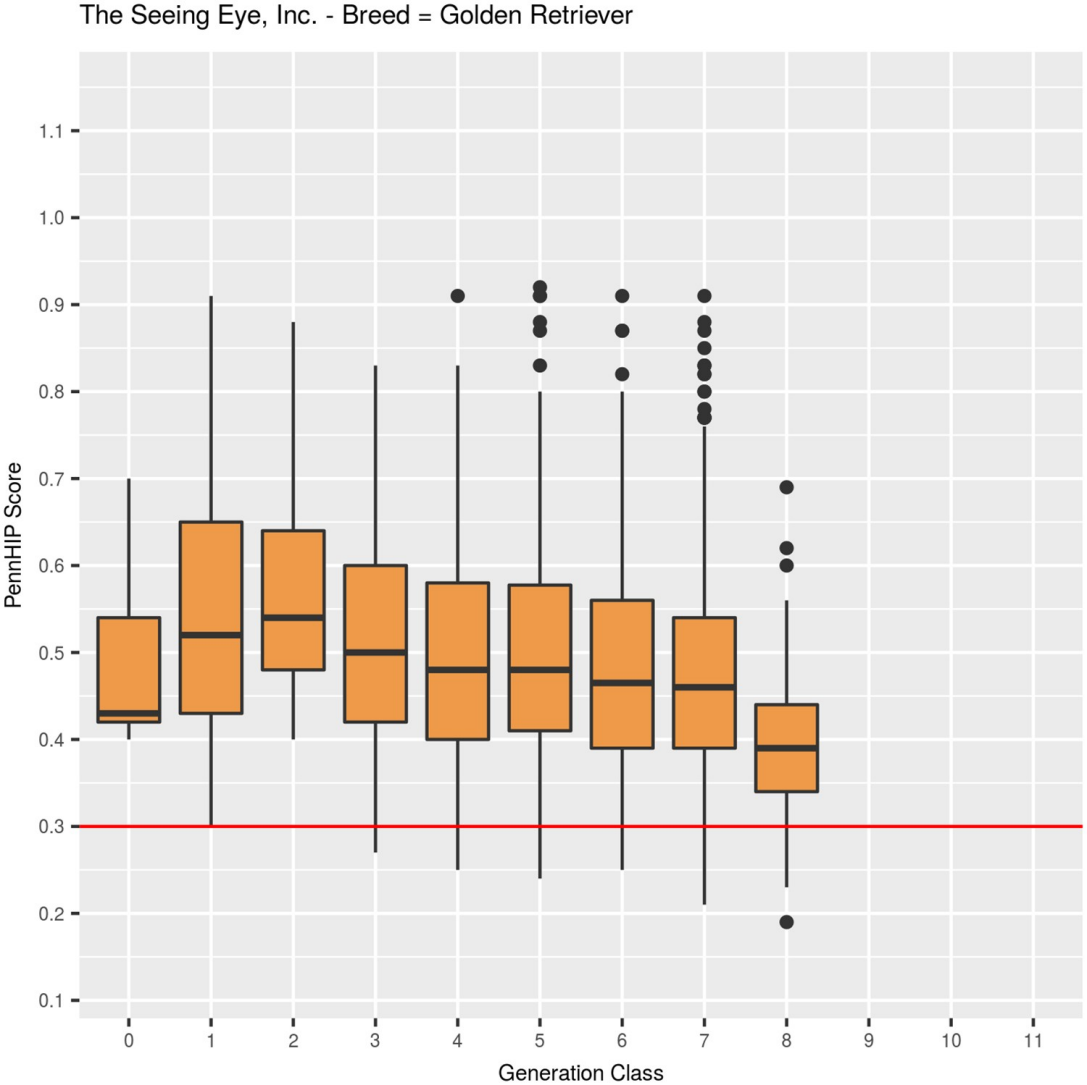
PennHIP distraction index values summarized across 9 generation classes for Golden Retrievers.

Average DI among 2,104 GRs was 0.487 (SEM = 0.003, SD = 0.1251), ranging from a minimum of 0.19 to a maximum of 0.92 (Table 2). Observed quartiles were: Q25 = 0.39, Q50 = 0.46, and Q75 = 0.56. The first generation GRs evaluated by DI were born in 1994, so this breed did not undergo a decade of HES selection prior to obtaining the first PennHIP scores, although as was the case with both GSDs and LRs, no DI values were used for making young breeder selection decisions prior to 1995. Among 41 GRs evaluated in generation 1 (Fig 8), average DI was 0.55. Only 13 dogs in generation 2 were evaluated, with mean DI of 0.58. In generation 3, the number evaluated increased to 106, with a mean DI of 0.51. In generation classes 4 and 5, mean DI held steady at 0.50, then decreased to 0.48 in generations 6 and 7. Among 116 GRs evaluated in generation 8, mean DI decreased to 0.40. Across generation classes, the change in mean DI values was highly significant, whether generation was fitted as a covariate (F_1,2100_ = 40.6, P<0.001) or as a classification effect (F_8,2093_ = 10.34, P<0.001).

**Fig 9:**
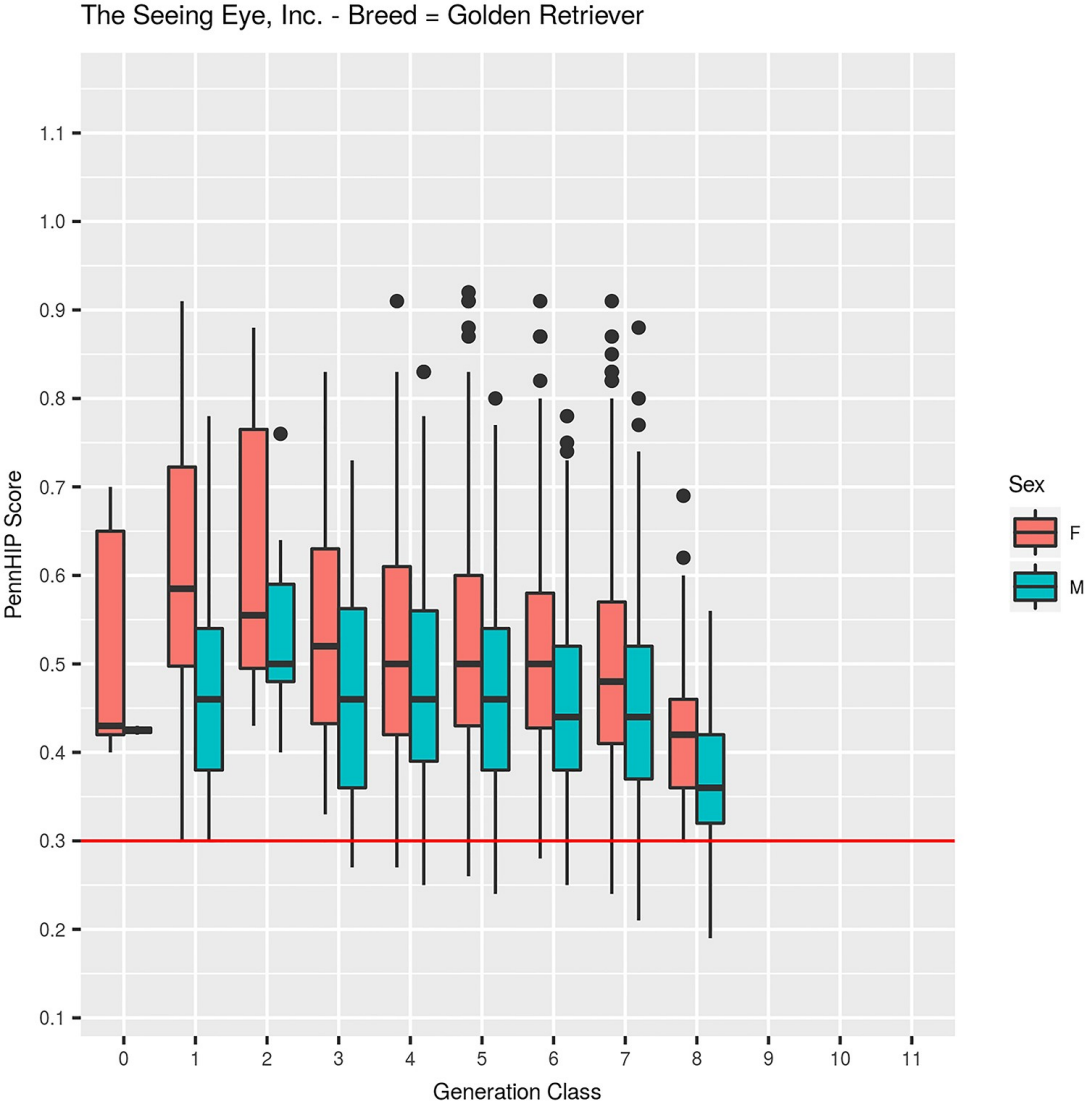
PennHIP distraction index values summarized by sex within generation class for Golden Retrievers.

Mean DI values differed between males (0.461, N = 1,106) and females (0.515, N = 998) by -0.054 DI units, which was a highly significant (F_1,2093_ = 108.2, P<0.001) difference (Fig 9). Inbreeding coefficients among GRs ranged from 0 to 27.0%, with a mean of 5.2%, and quartiles of: Q25 = 0%, Q50 = 3.7%, and Q75 = 8.2%. As inbreeding increased by 1%, DI values, on average, declined 0.001 units, which was a highly significant (F_1,2093_ = 24.0, P<0.001) decrease.

### Heritability and phenotypic and genetic correlations

Obtained using both MTDFREML [40] and *bayz* [43], within breed estimates of the additive genetic and residual variance components, heritabilities, and their standard errors are shown in Table 3 for both HES and DI. Using MTDFREML, heritability estimates for HES are 0.81 for GSD, 0.74 for LR, and 0.42 for GR, and for DI, the estimates are 0.63 in GSD, 0.76 in LR, and 0.60 for GR. Using *bayz*, estimates of heritabilities obtained for HES are 0.76 in GSD, 0.72 in LR, and 0.41 in GR, while for DI, the estimates are 0.60 in GSD, 0.66 in LR, and 0.59 in GR. All heritability estimates are significantly greater than zero (CI or HPD in Table 3, P<0.05) as shown by all 95% confidence intervals or highest probability density intervals that do not include zero.

**Table 3:** Estimates of genetic and residual variances and heritability of both the hip extended score and PennHIP distraction index observed in 3 breeds of guide dogs. Also shown are estimates of genetic correlation between these two phenotypes.

Hip Extended Score									
Breed	Time Period	Estimation Method^a^	N	Genetic Variance	Residual Variance	Heritability	Standard Error Heritability	95% CI^b^/HPD^c^	
								Lower	Upper
German Shepherd Dog	All records	REML	5,201	2.625	0.626	0.81	0.027	0.76	0.86
	Records through 1995	REML	2,293	2.041	2.321	0.47	0.067	0.34	0.60
	All records	Bayesian	5,201	2.420	0.744	0.76		0.70	0.82
Labrador Retriever	All records	REML	4,987	1.766	0.634	0.74	0.038	0.67	0.81
	Records through 1995	REML	1,909	1.459	2.360	0.38	0.079	0.23	0.53
	All records	Bayesian	4,987	1.700	0.662	0.72		0.61	0.83
Golden Retriever	All records	REML	2,308	0.998	1.380	0.42	0.061	0.30	0.54
	Records through 1995	REML	370	1.144	2.322	0.33	0.171	-0.01	0.67
	All records	Bayesian	2,104	0.984	1.397	0.41		0.29	0.55
Distraction Index									
German Shepherd Dog	All records	REML	3,782	0.0062	0.0036	0.63	0.037	0.56	0.70
	Records through 1995	REML	903	0.0077	0.0061	0.56	0.078	0.41	0.71
	All records	Bayesian	3,782	0.0054	0.0036	0.60		0.53	0.66
Labrador Retriever	All records	REML	3,662	0.0148	0.0046	0.76	0.032	0.70	0.82
	Records through 1995	REML	605	0.0092	0.0159	0.36	0.116	0.13	0.59
	All records	Bayesian	3,662	0.0096	0.0048	0.66		0.59	0.74
Golden Retriever	All records	REML	2,104	0.0100	0.0066	0.60	0.056	0.49	0.71
	Records through 1995	REML	192	0.0134	0.0077	0.63	0.194	0.25	1.01
	All records	Bayesian	2,104	0.0097	0.0067	0.59		0.49	0.68
Genetic Correlation Between HES and DI									
				Genetic Covariance	Residual Covariance	Genetic Correlation	Standard Error of Genetic Corr	Lower 95% CI^b^/HPD^c^	Upper 95% Ci^b^/HPD^c^
German Shepherd Dog	All records	REML	5,201	-0.0777	-0.0115	-0.61	0.05	-0.70	-0.52
	Records through 1995	REML	2,293	-0.0937	-0.0238	-0.75	0.09	-0.93	-0.57
	All records	Bayesian	5,201			-0.28		-0.38	-0.18
Labrador Retriever	All records	REML	4,987	-0.1244	-0.0193	-0.77	0.03	-0.84	-0.70
	Records through 1995	REML	1,909	-0.0802	-0.1128	-0.69	0.15	-0.99	-0.39
	All records	Bayesian	4,987			-0.21		-0.32	-0.10
Golden Retriever	All records	REML	2,308	-0.0758	-0.0196	-0.76	0.06	-0.88	-0.64
	Records through 1995	REML	370	-0.0857	-0.0470	-0.69	0.27	-1.23	-0.15
	All records	Bayesian	2,104			-0.29		-0.35	-0.23

^a^REML = Restricted Maximum Likelihood

^b^95% confidence interval for the “REML” estimates.

^c^95% highest probability density interval for Bayesian estimates.

**Fig 10:**
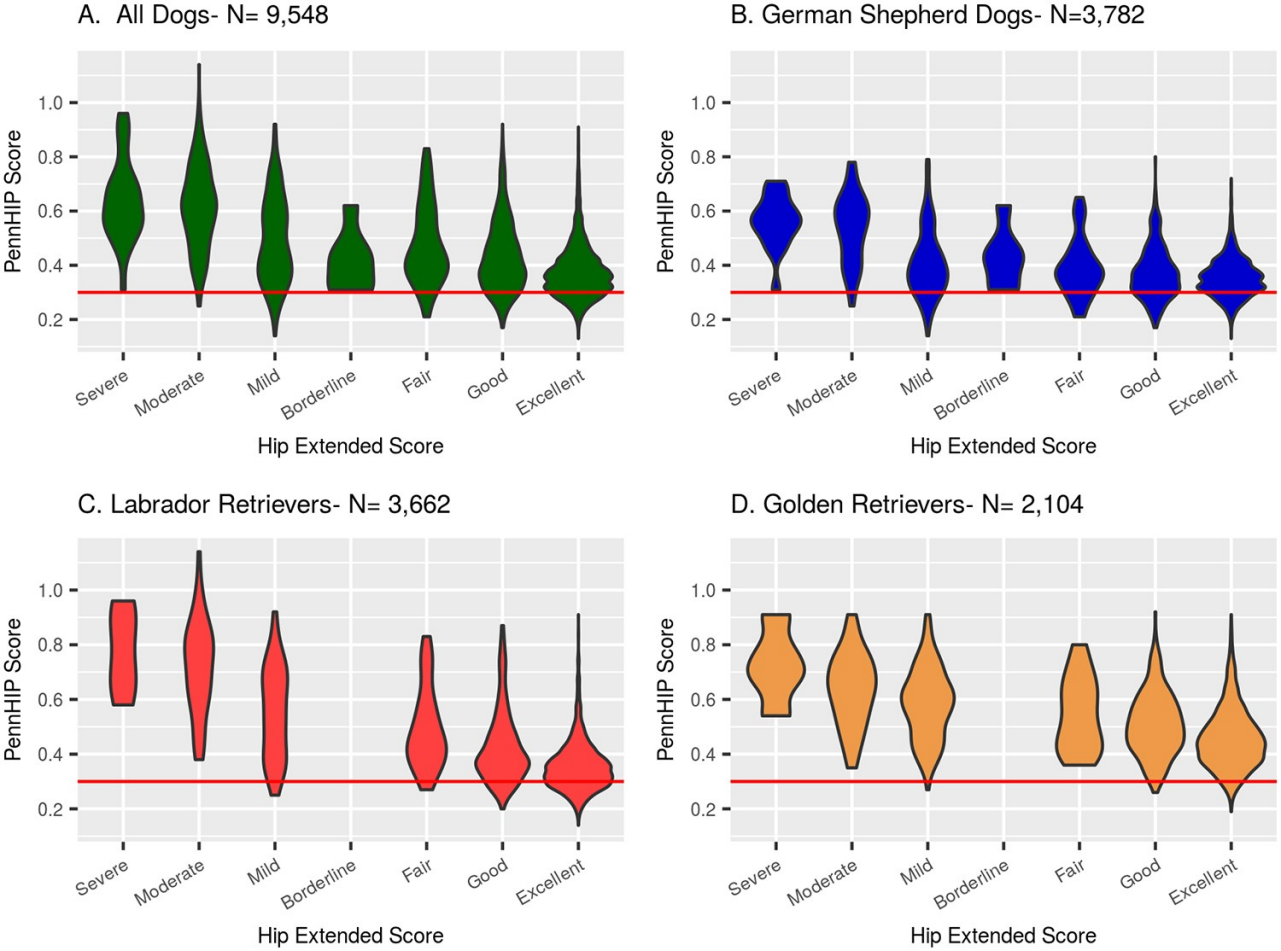
Distribution of DI values observed among dogs given each HESC. All dogs are shown in panel A, GSD are in panel B, LR are in panel C, and GR are in panel D.

Estimates of phenotypic correlation between the 2 measures of hip quality were: -0.314 (95% CI: -0.343 to -0.285) in GSD, -0.483 (95% CI: -0.508 to -0.458) in LR, and -0.41 (95% CI: -0.448 to -0.372) in GR. Estimates of genetic correlation between the 2 measures of hip quality in each of the 3 breeds (Table 3) obtained using MTDFREML were −0.61 for GSD, −0.77 for LRs, and −0.76 for GR. When estimated using *bayz* (Table 3), the genetic correlations were much lower at −0.28 in GSD, −0.21 in LR, and −0.29 in GR. The negative correlations reflect an inverse scoring scale between the two phenotypes, where the highest quality hips have low DI values and high HES. All phenotypic and genetic correlation estimates are significantly different (P<0.05) from zero, noted by observing that all 95% confidence intervals fail to include zero. A visual sense of the phenotypic relationship between HES and DI is shown in Fig 10, where the distributions of DI values observed among dogs given each HESC are summarized, both for all breeds combined and seperately by breed.

## Discussion

### Genetic correlation between HES and DI

Perhaps the most interesting finding in this report is in the estimates of genetic correlation between HES and DI reported in Table 3 for 3 breeds. The genetic correlations estimated by restricted maximum likelihood (REML) were approximately 3 times larger than those estimated using Bayesian methods, which begs the question: which ones are more likely correct? To develop an answer for this question, one can look at the correlated response expected in DI when direct selection is applied to HES. In theory, the negative genetic correlation between HES and DI suggests that selection for dogs with more desirable HES should, on average, result in dogs with lower DI values [52]. The magnitude of change in DI to expect by indirect selection on HES is determined by the degree to which genes controlling variation in HES also control variation in DI.

From basic quantitative genetic theory [36], the correlated response (CR) relationship can be expressed as:

$${CR}_{DI}\;=\;{i_{HES}h}_{HES}r_{A(HES,DI)}\sigma_{A(DI)}$$

where *i* = selection intensity with respect to HES, *h*_*HES*_ = square root of heritability for HES, *r*_*A(HES*,*DI)*_ = genetic correlation between HES and DI, and *σ*_*A*(*DI*)_ = genetic standard deviation of DI (square root of genetic variance of DI). Selection intensity is the selection differential divided by the phenotypic standard deviation. The selection differential is the difference between mean HES of dogs chosen to become parents and the overall mean of the population from which they were chosen [36].

Using the genetic parameters estimated in this study (Table 3) the correlated response in DI that could be expected based upon direct selection for improved HES is shown in Table 4 for 3 levels of selection intensity. If the genetic correlation is high, say -0.6 or larger, then one would expect selection for improved HES to produce a discernible decrease in DI. On the other hand, if the genetic correlation is weak, say -0.25 or less, then one would expect selection for improved HES to produce little or no improvement in DI. The truth of both of these statements, however, is predicated on the assumption that *i*, selection intensity, of HES is substantially greater than zero, indicating that sufficient selection pressure is being applied to produce some genetic change.

**Table 4:** Expected correlated response in DI based upon direct selection on HES.

Breed	Heritablilty (HES)	Genetic Correlation (HES, DI)	Genetic Variance (DI)	Correlated Response When i = 1	Correlated Response When i = 0.5	Correlated Response When i = 0.1
Restricted Maximum Likelihood Methodology						
German Shepherd Dogs	0.81	-0.61	0.0062	-0.043	-0.022	-0.004
Labrador Retrievers	0.74	-0.77	0.0148	-0.080	-0.040	-0.008
Golden Retrievers	0.42	-0.76	0.0100	-0.049	-0.025	-0.005
Bayesian Methodology						
German Shepherd Dogs	0.76	-0.28	0.0054	-0.018	-0.009	-0.002
Labrador Retrievers	0.72	-0.21	0.0096	-0.018	-0.009	-0.002
Golden Retrievers	0.41	-0.29	0.0097	-0.018	-0.009	-0.002

In the early generations of this study when variation in the HES metric still existed, selection pressure based on HES was being applied. As HES improved over successive generations, however, all dogs became phenotypically similar for HES, resulting in a decline in selection intensity. If the genetic correlation is high (REML estimates) and *i* = 1, indicating that the selection differential is 1 phenotypic standard deviation above the HES mean, the correlated genetic improvement in DI based on direct selection for improved HES was predicted to be -0.043 DI units in GSD, -0.080 DI units in LR, and -0.045 DI units in GR. In contrast, if the genetic correlations are low, like those estimated by Bayesian methods, then the correlated genetic improvement in DI expected from direct selection for improved HES is much lower at -0.018 DI units for all 3 breeds when *i* = 1. If *i* is only 0.5, the expected correlated change in DI is reduced by half, and if *i* is only 0.1, the expected change in DI is reduced to almost zero (Table 4). The actual correlated response in DI as a result of direct selection for improved HES observed in the early generations of this study strongly suggests that the genetic correlation between these two hip quality metrics is nearer to zero, not -1.0, giving support for the Bayesian estimates over the REML estimates.

There is one literature report [53] where an MTDFREML-derived estimate of genetic correlation between DI and an HEV-based score called EHR was found to be 0.69. This is opposite in sign but similar in magnitude to the MTDFREML estimates of genetic correlation found in the present study. The difference in sign arises because, in the literature report, smaller numbers reflected higher quality hips for both the DI and EHR score phenotypes. It needs to be noted, however, that this estimate of genetic correlation was based on hip quality measurements observed on 17 different breeds or breed crosses, which included GSD, LR, and GR along with an assortment of other breed classes, all of which were considered simultaneously in one analysis [53] by including breed composition as a fixed-effect term in the genetic model. To be appropriate, this approach presumes that the underlying genes producing genetic variation in measures of hip quality are the same genes affecting hip quality expression across all 17 breed classes. In that report [53], there was no suggestion that the authors tried using Bayesian techniques.

In a second literature report [54], estimates of genetic correlation obtained using REML methods were compared to estimates obtained using Bayesian methods. The REML-produced estimates from that study tended more often to diverge toward the edges of the parameter space at either +1 or -1, while the Bayesian-produced estimates remained in the intermediate regions. In that study, genetic relationships between pairs of animals were determined using genomic techniques based on chromosome markers, rather than pedigree-based relationships used in the present study, and the phenotypes being studied were in dairy cattle, not dogs, but the authors noted that REML-based methods were observed to sometimes encounter convergence issues.

Estimates of genetic correlation in the present study in the range between -0.30 and -0.25 suggest that only a small part of the genes affecting hip joint conformation as reflected by the HES are also affecting the expression of DI. If a correlated response in DI did not result from indirect selection for improved HES, then it is reasonable to ask if there is any evidence to support a direct selection response in DI? To answer this question, the actual selection differentials by generation class were tabulated in Table 5. It must be noted, however, that in the early generation classes, DI scores were not available at the time selection decisions were being made. Those scores only became available years later, so even though selection differentials are shown for early generation classes, they are retrospective, not prospective. Across all generation classes in all 3 breeds, all selection differentials were negative or zero, meaning that dogs chosen for breeding based on HES were equal to or had slightly tighter hips (lower DI) than the mean of their generation class, except for the second generation class in LR. In that class, the selection pressure applied by the HES corresponded to breeding dogs with more hip laxity (higher DI) than the mean of their generation class. This illustrates that even choosing parents based on HES EBVs sometimes produced offspring with higher DI’s, as seen in LR generations 3 and 4 (Tables 2 and 5) where mean DI actually increased (got worse), thus increasing risk of expressing hip OA with age among those puppies. This possibility has been previously reported [6] [27] [28] [55] and is again confirmed by this study.

**Table 5:** Selection differentials by generation class for PennHIP DI measured in 3 breeds of guide dogs. In early generation classes, the selection criterion for hip quality was based on HES. Both HES and DI were considered in the mid-range of generation classes, while only DI was considered in the latter generations.

	Parents		All Dogs		
Generation Class	Mean	N	Mean	N	Selection Differential
	German Shepherd Dogs				
1	Too few measured				
2					
3	0.36	21	0.37	213	-0.01
4	0.36	34	0.36	422	0.00
5	0.34	37	0.38	457	-0.04
6	0.36	32	0.37	512	-0.01
7	0.36	45	0.36	587	0.00
8	0.35	29	0.37	684	-0.02
9	0.36	30	0.37	521	-0.01
10	0.30	19	0.33	293	-0.03
11	0.28	1	0.32	12	-0.04
	Labrador Retrievers				
1	Too few measured				
2	0.44	9	0.38	39	0.06
3	0.36	28	0.42	294	-0.06
4	0.38	40	0.42	482	-0.04
5	0.36	40	0.40	546	-0.04
6	0.35	42	0.36	544	-0.01
7	0.34	42	0.36	711	-0.02
8	0.33	39	0.36	525	-0.03
9	0.32	23	0.33	395	-0.01
10	0.30	8	0.30	116	0.00
	Golden Retrievers				
1	Too few measured				
2					
3	0.46	15	0.51	106	-0.05
4	0.48	47	0.50	385	-0.02
5	0.47	29	0.50	510	-0.03
6	0.45	32	0.48	458	-0.03
7	0.43	21	0.48	468	-0.05
8	0.40	5	0.40	116	0.00

### Variation in DI remaining after selection for improved HES

With respect to hip quality, the long-term breeding goal is simple: produce puppies with no risk of developing OA as they age. Furthermore, when these young dogs reproduce, their puppies should also have low or no risk of developing OA. Such was the long-term goal of TSE’s breeding plan with respect to hip quality when it began in 1980. The premise at the outset was that selection for improved HES would lead to the production of puppies with low or no genetic risk of developing OA. Since 1980, however, evidence has accumulated [24] [56] [57] [11] [58] showing that even when both parents have an HEV-based Excellent phenotype at 12 or 24 months of age, some will still produce puppies at risk of developing OA as they age. With the 1980 premise in mind [59], it is instructive to examine the range in joint laxity observed among dogs intensely selected for improved HES.

**Fig 11:**
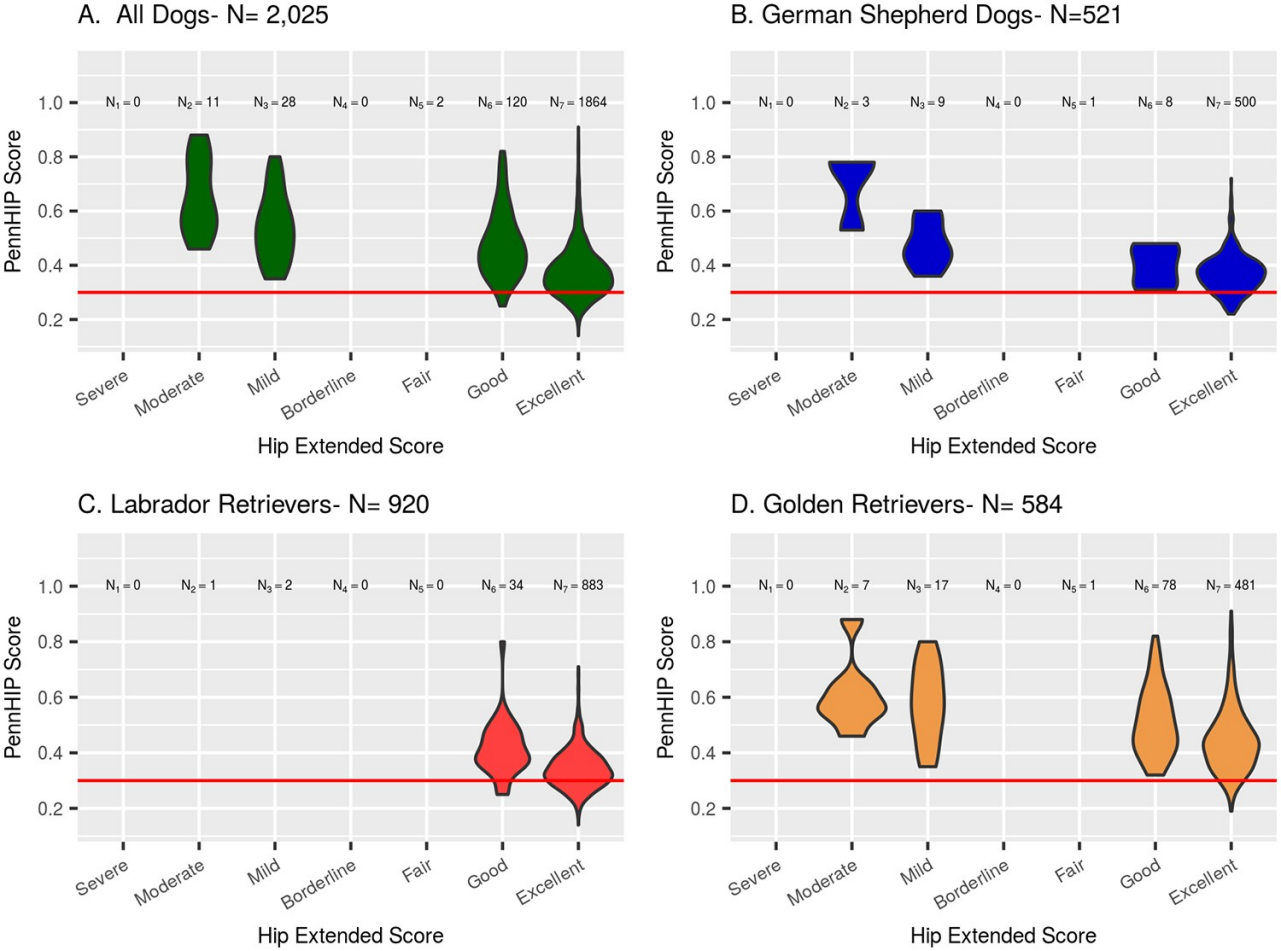
Distributions of DI values as a function of HESC class among dogs born into the latter generation classes. GSD’s born into generation 9 and later are in Panel B; LR’s born into generation 8 and later are in Panel C; and Golden Retrievers born into generation 7 and later are in Panel D.

By HESC class, the ranges in DI values are shown in Fig 11. GSD’s born into generation 9 and later are in Panel B; LR’s born into generation 8 and later are in Panel C; and Golden Retrievers born into generation 7 and later are in Panel D. All dogs included in Panels B, C, and D are combined into one graphic in Panel A. Among 2,025 dogs born into the latter generation classes, over 92% were graded as HESC Excellent. Among these, over 70% had DI values above 0.30, which placed them at increased risk of developing OA as they aged [6]. In Fig 11, it is easy to see that a substantial percent of the HESC Excellent dogs had DI values above 0.4 in all 3 breeds. This finding strongly suggests that the HESC phenotype is an inadequate metric for selecting against the OA of CHD produced by excess joint laxity [6] [60] [27].

Fortunately, the illustrations in Fig 11 clearly show that even among dogs with the HESC Excellent phenotype, there remains much variation in PennHIP DI values. The presence of phenotypic variation coupled with the high estimate of heritability for the PennHIP DI phenotype strongly implies that genetic improvement in hip quality could be rapidly achieved. Such a young breeder selection scheme could be simply implemented by choosing young dogs for breeding with PennHIP DI scores lower than their group mean. An even more rigorous approach would incorporate EBVs based on PennHIP DI scores into the young breeder selection process.

### Estimates of heritability

The estimates of heritability for HES found in the current study are much larger than the literature estimates generally reported for this phenotype [4] [24] [56] [57] [61]. They are, however, in close agreement with two literature reports [53] [33]. In the first study [53] referenced above in the discussion of genetic correlations, a group of 2,716 dogs were analyzed as one related population. They reported heritability estimates of 0.61 for DI and 0.76 for their HEV-based hip quality score [53]. The second literature report [33] used all OFA hip scores recorded between 1970 and 2015 where a breed reported 1,000 or more phenotypes evaluated. Estimates of heritability, obtained using Bayesian methodology, for the OFA hip score were reported as 0.59 for LR, 0.58 for GSD, and 0.65 for GR. These are the first estimates ever published using all hip scores recorded by OFA [33], not just the publicly available scores accessible on the OFA website.

In the current study, as time moved into the early 2000’s, almost all TSE-bred dogs were classified as having HESC Excellent or Good hips. The take-away message is that genetic selection had removed much of the phenotypic variation in HESC from generation 7 onward in GSDs and from generation 6 onward in LRs. Because the GR breeding population was substantially smaller and was added to the breeding program later than the other two breeds, the same loss of phenotypic variance was only beginning to occur in the 8^th^ generation class. The disappearance of phenotypic variation in any trait has a direct effect on the magnitude of the heritability estimate for that trait, as explained in detail below for HES.

As noted above for both GSDs and LRs, heritability estimates for HES (Table 3) obtained using either REML or Bayesian methods were more than twice as large as literature reported estimates [24] [56] [4] [57] [61] for similar hip quality scores derived from HEV radiographs when data across all years were analyzed. To understand why these heritability estimates were so much larger, the analyses were redone using REML [40] methodology, but including only records for dogs born through 1995. Estimates of genetic variances increased by more than 20% in both GSD and LR when all records were considered compared against the subset of early records observed through 1995, but the residual variance estimates were about 73% smaller (Table 3). This reflects the reality that few dogs received lower values for HES among GSDs born into generations 6 and beyond and LRs born into generations 4 and beyond. Since phenotypic variance is the denominator of the heritability ratio and phenotypic variance can be estimated as the sum of additive genetic and residual variance components, a smaller value for residual variance produced a larger estimate of heritability.

In GRs, only 370 HES records from 1995 and earlier were available for estimating heritability. Even so, the estimate of residual variance (2.322) was very similar to the estimates obtained (Table 3) for GSDs (2.321) and LRs (2.360), and as noted above for GSDs and LRs, the estimate of residual variance declined when all records were included in the analysis. Unlike GSDs and LRs, however, the decline in magnitude of the residual variance was only about 40%, instead of over 70%. In contrast to GSDs and LRs, the estimate of genetic variance for HES in GRs also declined when all records were analyzed. These changes in magnitude of the variance component estimates produced smaller changes in the heritability estimates for HES in GRs than was observed in GSDs or LRs. The trend remained, however: As genetic selection continued to produce improvement in the HES metric, phenotypic variation declined. This suggests that GRs will soon become phenotypically identical with respect to the HES metric, like has already happened in GSDs and LRs, thus rendering the HES a no longer useful metric to continue to improve hip quality.

TSE’s success in genetically improving HES may be attributed to: (1.) the use of EBVs, and (2.) the methodical recording of HES on each dog old enough to be evaluated. EBVs are a more accurate selection criterion for any polygenically inherited phenotype, but they are especially useful for identifying genetically superior young breeding candidates when heritability of the phenotype is smaller. This is true because EBVs properly weight information from relatives according to their degree of relationship to the dog in question, while also taking into account the magnitude of heritability. Thus EBVs pull together into a single number for each dog an array of information, both from the phenotype of the individual dog and from the phenotypes obtained on each of a dog’s relatives. This explains why EBVs are a more accurate method of comparing dogs evaluated for a particular phenotype in contrast to mass selection, where only a dog’s own phenotype is used as the basis for comparison.

Unfortunately, most private dog breeders and many working dog breeding organizations around the world simply do not have access to EBVs calculated on all dogs being considered as breeding candidates, although this is slowly beginning to change. There are, for example, hip quality improvement programs in Finland [62], Germany [63], and Australia [64] that calculate EBVs for HEV-based hip quality scores, and some of these are now making this EBV information available to private dog breeders [63] [64]. Among hip quality improvement program literature reports [24] [33] [35], none demonstrated a similar marked improvement to the improvement reported for the TSE populations using only mass selection driven by HEV-based hip quality score phenotypes. A primary reason for this lack of meaningful improvement stems from the fact that most hip quality registries worldwide are seriously flawed by selective submission bias [65]. Registries that permit dog owners or their veterinarians to selectively refuse to submit records for dogs with the worst hips make it impossible for even EBVs to sort out which are the best young dogs to be kept for breeding.

**Fig 12:**
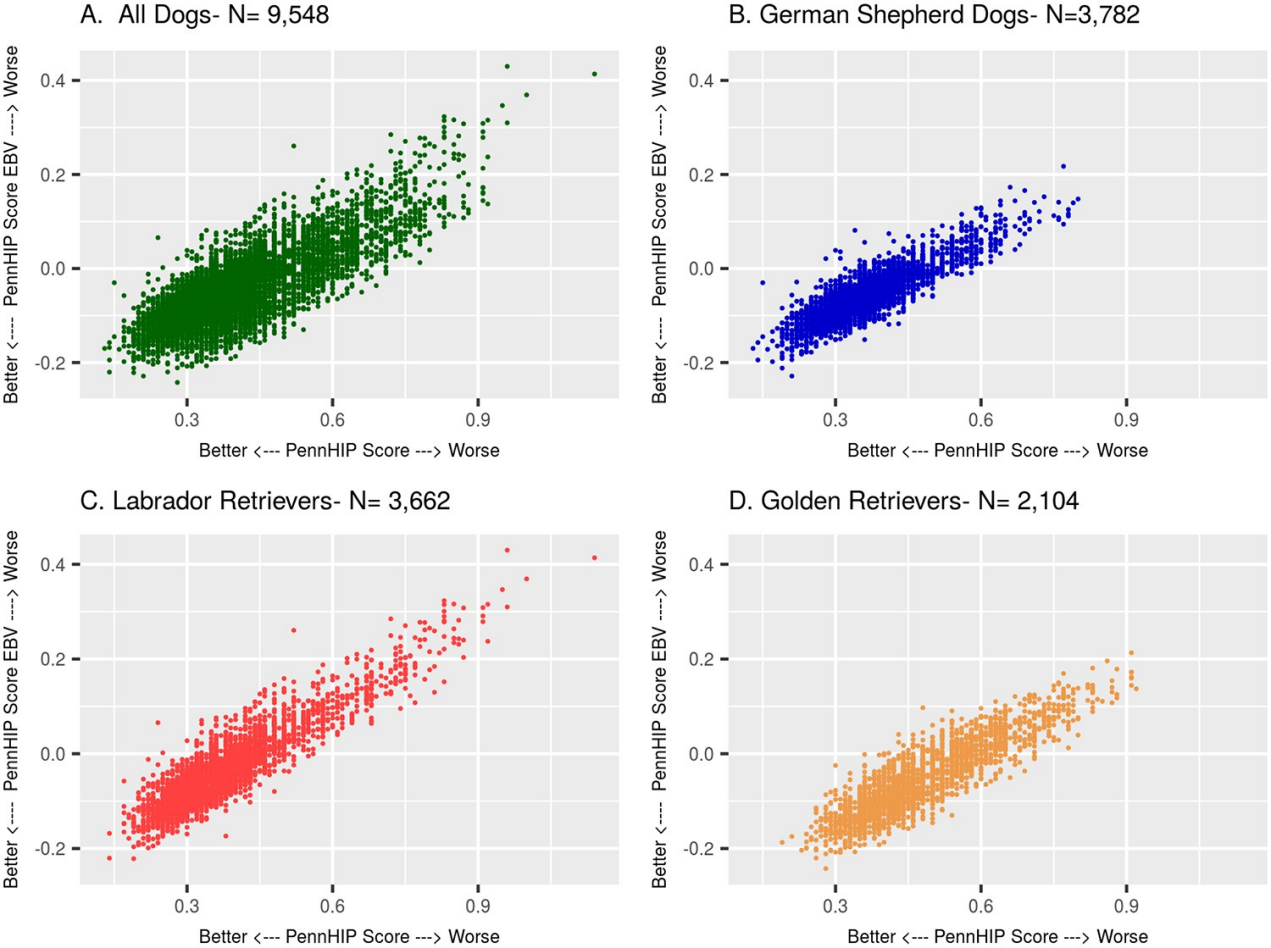
Scatterplot of estimated breeding values for PennHIP DI versus each dog’s own observed PennHIP DI value. All dogs in the study are shown in Panel A. Within breeds are shown in Panel B for GSD, C for LR, and D for GR.

The impact of lack of access to EBVs for making young breeder selection decisions is illustrated in Fig 12 where PennHIP DI EBVs are plotted on the y-axis versus each dog’s own PennHIP DI on the x-axis. In Panel A (Fig 12) are plotted the EBV–DI pairs for 9,548 dogs of all 3 breeds. The EBVs were estimated on a within breed basis, then combined into one plot in Panel A only for illustration. The single breed plots are shown in Panels B, C, and D for GSD, LR, and GR, respectively. The point regarding the utility of using EBVs as the selection criterion is easily seen by looking at the 0.30 point on the x-axis, although the point can be made using any value on the x-axis. Among dogs with a DI phenotype of 0.30, there was a range of PennHIP DI EBVs observed on the y-axis, from a low of about —0.20 DI units to a high of about +0.03 DI units. The interpretation of values observed over this range of EBVs is straightforward: not all dogs with an observed PennHIP DI of 0.30 are genetically equal. This means that some dogs with the 0.30 phenotype are predicted to possess more genes for tighter hips than other dogs with the same 0.30 phenotype. If a dog with -0.20 DI EBV is allowed to reproduce, it will likely yield puppies with very low DI values. If instead, the dog chosen for breeding is the one with a DI EBV of +0.03 DI units, it is likely to produce puppies with more joint laxity, and those puppies will be at increased risk of developing OA as they age. This fundamental difference in genetic worth as a young breeder cannot be discerned by looking only at the DI phenotype, but it is revealed by comparing dogs using EBVs.

The data summarized in Panel A can also be interpreted in a different way by looking at a horizontal line drawn at y = -0.10. Moving along that line, the first green dot appears at about the 0.16 point on the x-axis, which means that the dog observed to have the genetically best value on the y-axis at y = -0.10 had an observed DI value of 0.16, which is a very tight hip score on the PennHIP DI scale. Looking further to the right along the y = -0.10 line, however, shows that dogs with an equal genetic potential for producing puppies with tight hips were observed to have PennHIP DI values as high as about 0.50. Dogs with phenotypic PennHIP DI values as high as 0.50 would not likely be considered for breeding without also knowing the DI EBV and how to interpret it.

If one has only PennHIP DI phenotypes to use for selecting young replacement breeding dogs, then choosing ones with lower DI values is, on average, likely better than choosing ones with higher values. If the PennHIP DI values have, however, been used to calculate EBVs, then choosing young dogs for breeding with lower DI EBVs will likely be superior to any other approach for selecting young breeding stock. The point then is clear: EBVs help put into clearer perspective the genetic breeding potential of a dog regardless of the actual phenotype observed on that particular individual animal. This also brings into sharp focus an equally important, but often overlooked point: EBVs are only of value when available on a population of animals. It is only in the context of a population of values that a single EBV becomes meaningful. Knowing the EBV of a single dog, without any knowledge of the population of values from which that single value arose, is worthless.

#### Changes in TSE hip quality assessment over time

From the late-1970’s through the 1980’s, veterinary orthopedic and radiology specialists were looking at joint laxity in the young dog as a potential risk factor for developing OA [4] [23]. In the mid-1980’s, TSE agreed to participate in exploratory studies being done by the University of Pennsylvania comparing a new measure of joint laxity to the information being derived from HES. The first TSE-bred dog radiographed in both the distraction and compression views occurred in September 1987. An additional 52 were radiographed in these views through 1990, and in 1991, TSE began routinely submitting both compression and distraction views on every dog along with HEV to the research project.

Through 1995, HES was the only hip quality measurement available to TSE. Using EBVs based only on the HES, measurable phenotypic improvement in that metric was apparent in 3 breeds in as few as 5 generations of selection (Table 1; Figs 1–3). Within a year after PennHIP DI measures became available in 1995, EBVs for the DI phenotype were being calculated and had been added to the overall selection index. For five years from 1996–2000, primary selection emphasis was being applied to improve other traits, although both HES and DI EBVs and phenotypes were used to apply some selection pressure on improving hip quality.

By 2001, further improvement of hip quality based solely on the HES phenotype was challenging because almost no selection pressure could be applied. This is illustrated in Table 1 and Figs 1 and 2, by noting that over 90% of all GSDs in generations 6 and later and LRs in generations 4 and later were CHD normal. Furthermore, as generations progressed, an increasing percent of dogs were scored as excellent. In other words, based upon the HES metric observed in later generations, all GSDs and LRs were identical in phenotypic hip quality, having either HESC Good or Excellent hips. Once all members of any population are identical for a phenotype, it is impossible to make further genetic change in that phenotype because no selection pressure can be applied [36] [66] [67]. In GR’s, over 80% were CHD normal by generation 5, and by generation 7, almost 95% were CHD normal with over 81% scoring excellent, suggesting that the genetic change in hip quality for this breed was progressing similarly to the path followed by GSDs and LRs, (Figs 1, 2 and 3).

In the years from 2001 to 2009, selection pressure for improving hip quality was relaxed even more because almost all dogs scored by HESC received Good or Excellent ratings. During those same years, other health conditions and behavior phenotypes dominated the list of traits needing to be genetically improved, however, hip quality monitoring continued using both HES and PennHIP DI phenotypes.

Selection for improved HES did reduce joint laxity, and that reduction was sufficient to impart genetic protection against OA for about half the TSE-bred dogs evaluated for hip quality. The remaining half or more, however, had DI values above 0.30, with those possessing higher DI values left at increasingly greater risk of developing OA with age. Evidence supporting this point comes from the hip quality results observed among latter generation dogs. Consider that TSE produced over 800 GSDs in generations 9 and beyond and over 1,000 LRs in generations 8 and beyond with 96% receiving an HESC Excellent hip score (Table 1, Figs 1 and 2). Even though selection for improved HES produced dramatic improvement in that phenotype, DI measures (Table 2, Figs 4 and 5, generation classes 3–9 in GSD; generation classes 3–8 in LR) indicate that direct selection for improved HES produced only small or no correlated change in DI, varying a bit by breed. In GSDs, for example, median DI among 213 dogs examined in generation 3 was 0.35. Five generations later, median DI was 0.36 (N = 684) in generation 8. Over those same generations, HESC changed from 46% (HESC = Fair, Good, or Excellent; N = 273) of dogs with CHD normal hips in generation 1 to almost 97% (N = 695) in generation 8. Furthermore, over 93% of GSDs in generation 8 were graded as having HESC Excellent hips, again illustrating the impossibility of applying additional selection pressure on HESC.

Over 7 generations of LR selection based primarily on the HES metric, dogs with normal hips (HESC = Fair, Good, or Excellent) increased from 59% (N = 323, generation 1) to 99% (N = 717, generation 7) as shown in Table 1 and Fig 2. Measurement of DI values began on a meaningful number of LRs in generation 2 (Table 2, Fig 6), where median DI was 0.36 (N = 39). In generation 3, median DI actually worsened to 0.38 (N = 294), and it remained at that level in generations 4 (N = 482) and 5 (N = 546). In generation 6, however, it declined to 0.35 (N = 544) where it remained in generations 7 and 8 (N = 711, and 525, respectively). The overall conclusion for LRs is that even when HESC improved dramatically, to the point where over 94% had Excellent hips in generation 8, fewer than 25% in that generation class had DI values below 0.30 (Table 2).

Among GRs, dogs with normal HEV phenotype (HESC = Fair, Good, or Excellent) increased from 67% (N = 51, generation = 1) to 95% (N = 473, generation = 7) over 7 generations of selection, although fewer GRs scored HESC Excellent and more scored Good or Fair than was seen in either GSDs or LRs (Table 1, Fig 3). In generation 1, median DI was 0.52 (N = 41), then in generation 2, it increased to 0.54 (N = 13), after which it dropped to 0.50 in generation 3 (N = 106, Table 2, Fig 8). From generations 4 to 7, median DI decreased to 0.48 for 2 generations, then to 0.47 in generation 6 and to 0.46 in generation 7. Even though average DI values declined, the distribution of values among generation 7 GRs shows the first quartile for DI as 0.39, indicating that well over 75% of GRs in that generation class were at elevated risk of developing OA with age since they had DI values greater than the 0.30 threshold [6] [27].

In the latter generation classes, as an increasing number of dogs received HESC Excellent, the HES was no longer a useful metric for hip phenotype screening because it no longer provided actionable information regarding relative genetic merit of hip quality. To illustrate this point, compare the distribution of HES among generation 1 LR offspring, where 59% (N = 323) had a “normal” HES, to those in generation 10, where 100% (N = 116) received HES Excellent (Fig 2). The initial range in HES of dogs in generation 1 allowed application of selection pressure, i.e. choosing breeding candidates with scores better than the population mean. By generation 10, however, no variation in HESC remained, thus making it impossible to apply any selection pressure on this trait. Variation in PennHIP DI, even though it was declining, continued to provide actionable information in all three breeds thus enabling the application of selection pressure for improving hip quality. For a working dog organization like TSE, which has reached a steady state where all dogs evaluated have the most preferred HESC Excellent phenotype, the PennHIP DI provides a highly heritable alternative metric of hip quality.

In 2010, it became clear that the application of direct selection pressure to reduce DI would be required to move mean DI lower. Evidence that mean DI values are moving lower is found in the last 1 or 2 generation classes of each breed (Table 2, Figs 4, 6, and 8). In GSDs, median DI in generation 10 declined to 0.33 (N = 293) down from 0.37 (N = 521) in the previous generation. Even though only 12 GSDs in generation 11 had been evaluated, their median DI was 0.30. In LRs, median DI of 395 dogs in generation 9 was 0.32, down from 0.35 the previous generation, and among those in generation 10 (N = 116), median DI declined further to 0.30. This means that half the LRs in generation 10 are at very low risk to develop OA in their lifetime. In GRs, median DI in generation 8 was 0.39 (N = 116), and even though that is a relatively high DI mean, it is a biologically meaningful decline from the 0.46 median DI observed in generation 7 (N = 468).

### Limitations in the current study

There are limitations to the current study. The first and most important limitation is that while it was demonstrated that TSE achieved genetic improvement in the HES, it must be emphasized that hip screening occurred at an average age of 15.6 months. The diagnosis of CHD has been shown to be age-dependent [20] and the onset of hip OA has been shown to be linear with age [60] [68]. Therefore, while these dogs did not have OA at 15.6 months of age, it is likely that more dogs would have shown evidence of OA if evaluated at 24 months of age, as is the common practice in the USA, or later in life. A lifespan study following the hip phenotype of 48 LR, all susceptible to OA based on DI, demonstrated that 98% of dogs developed hip OA by the natural end of life (average age at death 12.1 years) [60]. If applicable to the current investigation, 50% of TSE LRs in the last generation would be susceptible to hip OA (DI>0.3), despite 8 generations of selection based on EBVs from the hip-extended phenotype. It is probable that some or all of these dogs would develop hip OA later in life, possibly affecting their comfort, performance and work longevity. This leads to a second limitation of this study; one related to “clinical relevance” of the findings.

Data were not available from TSE records to determine how the HES or DI relates to the development of clinical signs, such as pain and lameness, or diminished work longevity. Unfortunately, such data are not systematically collected by TSE owing to the wide-spread locations of dogs across North America and the overwhelming manpower and expense that would be necessary to obtain such information. Evidence from the life-span study of LR [60], however, showed that dogs with lower DI’s (DI of 0.36–0.40) got OA later in life [11] and therefore would presumably show associated clinical signs later in life. The first Labrador retriever in that study to require analgesics for overt pain was 6.8 years of age [69]. Also, in that study, the HEV-based hip quality score in 2 year old dogs misdiagnosed as normal 58% of the dogs that showed OA later in life, and those authors concluded that the HEV-based score was a poor predictor of both OA and the potential for clinical signs [11].

A third limitation is that the effect of either body weight or body condition scores on HES was not evaluated in this study. Both effects have been shown in other studies to affect some HEV-based hip quality scores, but not PennHIP DI [68] [70].

## Conclusions and Key Findings

1. Improvement in hip quality can be achieved by selection based on subjective scoring of the HEV radiograph when ALL dogs are evaluated and the recorded scores are combined to produce EBVs. EBVs, rather than individual hip scores, should then be used to select breeding dogs.
2. In the latter generations of this study, hip improvement based on HES selection has practically reached an endpoint for all three breeds, that being Excellent hip phenotype. In the last generation class of each breed containing more than 100 dogs, 99% had HESC Excellent hips.
3. When all dogs have nearly the same hip phenotype, almost no selection pressure can be applied to improve hip quality using the HES or HES EBVs.
4. In 3 breeds, 50% or more of dogs possessing the HESC Excellent phenotype born into the 8^th^ generation class or later remained at risk of developing OA before end of life as evidenced by them having PennHIP DI values above 0.30.
5. The PennHIP DI was found to be highly heritable in the three breeds in this study: German Shepherd Dogs, Labrador Retrievers, and Golden Retrievers.
6. The heritability of DI is large enough that “mass selection” (breeding based solely on the individual dog’s own DI value) would be adequate to make genetic improvement in hip status.
7. Calculating EBVs for the PennHIP DI could increase the accuracy of identifying young breeder candidates most likely to produce offspring with low risk of developing OA as they age. This could result in even faster genetic improvement of hip quality by eliminating mistakes made in choosing young dogs as breeders.
8. Estimates of genetic correlation between HES and PennHIP DI phenotypes were small in magnitude in all 3 breeds. This strongly suggests that genes controlling expression of joint laxity have only slight overlap with genes that control expression of hip joint conformation as seen in the HEV radiograph.
9. The PennHIP DI is an accurate hip-screening test that can be used now to help breeders make evidence-based young-breeder selection decisions. PennHIP phenotypes are collected with the aim of avoiding submission bias. PennHIP policy mandates that all PennHIP radiographs must be submitted for evaluation irrespective of laxity or osteoarthritis present. By measuring maximum passive hip joint laxity, PennHIP testing provides valid hip quality status information in young dogs. It is recommended that dog breeders and their veterinarians avail themselves of the PennHIP method to identify superior young animals to be kept for breeding following principles of mass selection. Incorporating the PennHIP distraction index into calculations of EBVs will, however, make even more rapid improvement in hip status of future generations of dogs.

## Supporting information

S1 File Data dictionary describing columns in data (S2 File) and pedigree (S3 File) files. (TXT)

S2 File Data analyzed for this report. (CSV)

S3 File Pedigree relationships among all dogs in this study. (CSV)
